# Molecular identification and probiotic potential characterization of lactic acid bacteria isolated from the pigs with superior immune responses

**DOI:** 10.3389/fmicb.2024.1361860

**Published:** 2024-03-21

**Authors:** Wenjie Ma, Wenli Zhang, Xinrong Wang, Yu Pan, Mengjie Wang, Yunfei Xu, Junxin Gao, Hongyu Cui, Changwen Li, Hongyan Chen, He Zhang, Changyou Xia, Yue Wang

**Affiliations:** ^1^State Key Laboratory for Animal Disease Control and Prevention, Harbin Veterinary Research Institute, Chinese Academy of Agricultural Sciences, Harbin, China; ^2^Heilongjiang Provincial Key Laboratory of Laboratory Animal and Comparative Medicine, Harbin Veterinary Research Institute, Chinese Academy of Agricultural Sciences, Harbin, China; ^3^College of Veterinary Medicine, Southwest University, Chongqing, China; ^4^National Center of Technology Innovation for Pigs, Chongqing, China

**Keywords:** lactic acid bacteria, immune responses, probiotic characteristics, safety assessment, antimicrobial activity

## Abstract

Lactic acid bacteria (LAB) belong to a significant group of probiotic bacteria that provide hosts with considerable health benefits. Our previous study showed that pigs with abundant LAB had more robust immune responses in a vaccination experiment. In this study, 52 isolate strains were isolated from the pigs with superior immune responses. Out of these, 14 strains with higher antibacterial efficacy were chosen. We then assessed the probiotic features of the 14 LAB strains, including such as autoaggregation, coaggregation, acid resistance, bile salt resistance, and adhesion capability, as well as safety aspects such as antibiotic resistance, hemolytic activity, and the presence or absence of virulence factors. We also compared these properties with those of an opportunistic pathogen EB1 and two commercial probiotics (cLA and cLP). The results showed that most LAB isolates exhibited higher abilities of aggregation, acid and bile salt resistance, adhesion, and antibacterial activity than the two commercial probiotics. Out of the 14 strains, only LS1 and LS9 carried virulence genes and none had hemolytic activity. We selected three LAB strains (LA6, LR6 and LJ1) with superior probiotic properties and LS9 with a virulence gene for testing their safety *in vivo*. Strains EB1, cLA and cLP were also included as control bacteria. The results demonstrated that mice treated LAB did not exhibit any adverse effects on weight gain, organ index, blood immune cells, and ileum morphology, except for those treated with LS9 and EB1. Moreover, the antimicrobial effect of LR6 and LA6 strains was examined *in vivo*. The results indicated that these strains could mitigate the inflammatory response, reduce bacterial translocation, and alleviate liver, spleen, and ileum injury caused by *Salmonella typhimurium* infection. In addition, the LR6 treatment group showed better outcomes than the LA6 treatment group; treatment with LR6 substantially reduced the mortality rate in mice. The study results provide evidence of the probiotic properties of the LAB isolates, in particular LR6, and suggest that oral administration of LR6 could have valuable health-promoting benefits.

## 1 Introduction

In recent decades, the growing need for livestock products, which necessitates growth promotion and disease prevention, has resulted in the extensive utilization of antibiotics as feed additives for animals (Zhang et al., 2022). The frequent and excessive use of antibiotics has led to the increased incidence of bacterial resistance (Ali et al., 2022). Antibiotic resistance is a global issue and poses a significant threat to the health of both humans and animals (Mehdi et al., 2018). Probiotics are currently being considered as a viable substitute for antibiotics in preventing pathogen infections (Afolayan et al., 2017).

Lactic acid bacteria (LAB) are the most commonly used probiotics that include a wide range of genera (Vogado et al., 2018). They have been widely used since ancient times as a safety and effective way to improve digestion and nutrient absorption, eliminate toxic substances, enhance immunity, and protect host from pathogens (Landete, 2017; Miller et al., 2017; Alard et al., 2018; Jäger et al., 2018). The growing demand for antibiotic-free animal production has led to the gradual prohibition of antibiotics. LAB are currently widely used in animal feeds to regulate the balance of intestinal microbiota, inhibit the growth of pathogenic bacteria, and promote the richness and diversity of the gut microbiota (Li et al., 2020; Mao et al., 2020).

The intestine is a crucial source of probiotic strains (Bai et al., 2020). Probiotics isolated from the human intestine, such as *L. gaseri*, *L. reuteri*, and *L. fermentum*, have demonstrated beneficial therapeutic and protective effects (Fontana et al., 2013). The pig intestine is also regarded as a viable source of probiotics (Petrof, 2009). Zhang et al. have revealed that *L. reuteri ZJ617*, which is isolated from pig intestines, is effective in combating pathogens such as *Escherichia coli* K88 and *Salmonella enteritidis* 50,335 (Zhang et al., 2013b). Our previous study showed that a higher abundance of LAB in pig gut microbiota was closely associated with increased antibody titer, reduced pathogenic damage, and shortened viral clearance time (Zhang et al., 2021). Isolating LAB with superior properties and the ability to regulate the immune response from pigs with stronger immune responses is highly probable, since these intestinal LAB have the potential to exist preferentially and settle easily in pig intestines. Therefore, the purpose of this study was to isolate LAB from pigs with high antibody titers and high LAB abundance and evaluate their probiotic potential and safety.

## 2 Materials and methods

### 2.1 Bacterial strains and cells

De Man, Rogosa, and Sharpe (MRS) broth (Hope Bio-Technology Co., Ltd., Qingdao, China) was used as the culture medium for isolating LAB. All pathogenic bacterial strains were stored in our laboratory. The pathogens *E. coli* (ATCC 8379), *Salmonella typhimurium* (ATCC 14028), and *Staphylococcus aureus* (ATCC 25923) were used to evaluate the coaggregation and bacteriostatic activities of LAB. *Streptococcus equi* (ATCC 33398) was used as positive controls for hemolytic activity. Brain Heart Infusion (BHI) medium used as the culture medium for the pathogenic strains. *Lactobacillus acidophilus* (ATCC 4356) and *Lactobacillus plantarum* (ATCC 8014) were selected as reference strains with probiotic properties, whole-genome sequencing, and wide use in lactic acid bacteria comparison (Vahedi Shahandashti et al., 2016; Gheziel et al., 2019). Caco-2 cells (an immortalized cell line derived from human colorectal adenocarcinoma) were cultured in Minimum Essential Medium (MEM, Gibco, United States) supplemented with 10% fetal bovine serum (FBS, Gibco, United States). HT-29 cells (a human colon cancer cell line) were cultured in RPMI 1640 (Gibco, United States) supplemented with 10% FBS. The Caco-2 and HT-29 cell lines were incubated at 37°C with 5% CO_2_.

### 2.2 Isolation of LAB

Fresh fecal samples were collected from Specific Pathogen-Free (SPF) pigs that had a high antibody titer against PRRSV and high abundance of LAB (Zhang et al., 2021), and immediately transported to the laboratory at 4°C. LAB of fecal samples were isolated and grown overnight in MRS medium. Then, they were spotted onto MRS agar plates. The LAB isolates were incubated at 37°C for 24–48 h. The genomic DNA of LAB was extracted using a bacterial genome extraction kit (Tiangen, China) according to the manufacturer’s protocols. The isolates were identified based on the 16S rDNA gene using the primer pair: 16S rDNA-F (5′-AGA GTT TGA TCC ATG GCT CAG-3′)/16S rDNA-R (5′-AAG GAG GTG ATC CAG CC-3′) (Bin Masalam et al., 2018). The 16S rDNA sequences were used to identify the bacterial species based on BLAST analysis.

### 2.3 Probiotic characteristics of LAB isolates

#### 2.3.1 Antimicrobial activities

The antimicrobial activities of the LAB isolates were analyzed using the agar plate diffusion method as previously described with slight modifications (Gómez et al., 2016). Individual isolates of LAB were cultured in MRS broth under aerobic incubation at 37°C for 48 h. After incubation, the LAB cells were separated by centrifugation at 4000 × *g* for 10 min, and the resulting liquid portion (supernatant) was then sterilized by filtration using syringe filters (0.45-μm). *E. coli, S. typhimurium*, and *S. aureus* were cultured overnight, washed three times, and resuspended in PBS to a final concentration of 10^7^ cells/mL. These bacterial suspensions were then evenly coated on LB agar plates at 100 μL per plate and air-dried at room temperature. Subsequently, wells (6 mm depth, 7 mm diameter) were carefully cut into the agar, and 100 μL supernatant was added to each well. Fresh MRS broth was used as a negative control. The culture plates were allowed to diffuse for 4 h at room temperature, followed by incubation at 37°C for 20 h. Finally, the diameters of the inhibition zones around the wells were measured.

#### 2.3.2 Autoaggregation and coaggregation assays

Autoaggregation assays were performed following the method described by Collado et al. (2007), with some modifications. Briefly, the LAB isolates were cultured in MRS medium at 37°C for 48 h. After incubation, the cultures underwent centrifugation at 5000× g for 10 min, followed by three washes and resuspension in 2 mL of PBS to an optical density (OD600) of 0.25 ± 0.05. The bacterial solution was then incubated at room temperature, and then OD values were measured at 0, 2, 4, 6, 10, and 24 h. At each time point, 100 μL aliquots were collected, and the absorbance (A) was measured at 600 nm. Autoaggregation rates were determined as [(AX – Ay)/Ax] × 100, where Ax represents the absorbance at time t = 0, and Ay represents the absorbance at t = 2, 4, 6, 10, or 24 h.

Coaggregation assays were performed by mixing 2 mL of each isolated strain and three pathogenic strains and incubated them at room temperature. The absorbance of the mixture was measured at 0, 2, 4, 6, 10, and 24 h. The coaggregation percentage was calculated using the following formula: [(Apro + Apat)-Amix]/(Apro + Apat) × 100, where Apro + Apat represents the absorbance of the mixture of the LAB and the pathogen at time 0 h, and Amix denotes the absorbance of the LAB mixture and the pathogen at different time points (Valeriano et al., 2014).

#### 2.3.3 Acid and bile salt resistance assays

The resistance of the LAB strains to acid and bile salts was assessed using a method previously described by Dowarah et al. (2018). The LAB strains were cultured overnight, harvested at 5000 × g for 10 min at 4°C and washed in PBS three times. The LAB strains were then resuspended in MRS broth with 0.3% (w/v) bile salts (Solarbio, China) or MRS broth (pH = 3) to a final concentration of 10^8^ cells/mL and incubated at 37°C for 2 h or 4 h. The number of LAB was counted at the initial (T0), 2 h (T2), and 4 h (T4) time points using the dilution plate coating method. The acid and bile salt resistance values were determined by calculating the number of surviving bacteria after incubation.

#### 2.3.4 Adhesion assay

The Caco-2 and HT-29 cells were plated into 24-well plates. Bacterial pellets from an overnight culture were washed three times in PBS and resuspended to a concentration of 10^8^ CFU/mL in PBS with fluorescein isothiocyanate (FITC) (100 μg /mL) and incubated at 37°C for 1 h in the dark. The non-adherent bacteria were washed with PBS three times to remove them. The cell monolayers were washed three times with PBS as well. Each well was then seeded with 500 μL of bacterial suspension (10^6^ CFU/mL) and the fluorescence intensity was measured using a microvolume spectrophotometer (PE Envision, United States). The plates were incubated at 37°C in 5% CO_2_ for 1 h and then the wells were gently washed with PBS five times to remove the unattached bacteria. Then, 0.1 mL of trypsin–EDTA was added to each well and incubated for 10 min at 37°C. Finally, cell culture medium with 10% FBS was added to stop digestion and the fluorescence intensity was measured. The adhesion rate was calculated using the following formula: Cell adhesion rate (%) = A/A0 × 100%, where A0 and A denote the fluorescence intensity before and after adhesion, respectively.

### 2.4 *In vitro* safety evaluation of LAB isolates

#### 2.4.1 Antibiotic susceptibility

The antibiotic susceptibility of the selected strains was assayed with modifications, as previously described (Shi et al., 2019; He et al., 2021). Briefly, overnight cultures (10^8^ CFU/mL) were spread on MRS plates at 100 μL per plate. The seeded agar medium was placed with commercial antibiotic discs (Binhe Microbial Reagent Co., Ltd., Hangzhou, China), containing oxacillin, penicillin, erythromycin, spectinomycin, clarithromycin, vancomycin, clindamycin, chloramphenicol, tetracycline, minocycline, norfloxacin, ciprofloxacin, ofloxacin, polymyxin B, Sulfamethoxazole, and Nitrofurantoin. The plates were incubated at 37°C overnight, and then the zone of inhibition (in mm) around the antibiotic disc was measured. The antibiotic susceptibility of the LAB strains was assessed according to Clinical and Laboratory Standards Institute (CLSI) guidelines (Cockerill, 2012).

#### 2.4.2 Hemolytic activity analysis

The LAB isolates were inoculated in 2 mL of MRS medium and cultured at 37°C for 24 h. Each of the LAB isolates was streaked on Columbia agar plates supplemented with 5% sheep blood and then incubated at 37°C for 24 h to assess the hemolytic activity. The blood agar plates were examined for signs of β-hemolysis (clear zones around colonies), α-hemolysis (green halo around colonies), or γ-hemolysis (no zones around colonies) (Talib et al., 2019).

#### 2.4.3 Detection of virulence genes

The isolates were tested by PCR for the presence of virulence genes, including aggregation (*asa1*), gelatinase (*gelE*), cytolysin (*cylA*), enterococcal surface protein (*esp*), hyaluronidase (*hyl*), accessory colonization factor (*ace*), endocarditis antigen (*efaA*), tyrosine decarboxylase (*tdc*), ornithine decarboxylase (*odc*) and histidine decarboxylase (*hdc*). The DNA of *E. faecalis*, whose genome contains all the virulence genes, was used as the positive control. The primers and annealing temperatures used for PCR are provided in Table 1.

**Table 1:** PCR primers and annealing temperatures used for the detection of genes implicated in the virulence of LAB isolates.

Virulence factor	Genes	Primer sequence (5′ → 3′)	Amplicon size (bp)	Annealing Tm (°C)	References
Aggregation substance	asa1	GCACGCTATTACGAACTATGA	375	56	Vankerckhoven et al. (2004)
		TAAGAAAGAACATCACCACGA			
Gelatinase	gelE	TATGACAATGCTTTTTGGGAT	213	50	Al-Talib et al. (2015)
		AGATGCACCCGAAATAATATA			
Cytolysin	cylA	ACTCGGGGATTGATAGGC	688	60	Vankerckhoven et al. (2004)
		GCTGCTAAAGCTGCGCTT			
Enterococcal surface protein	esp	GCTGCTAAAGCTGCGCTT	510	60	Al-Talib et al. (2015)
		AATTGATTCTTTAGCATCTGG			
Hyaluronidase	hyl	ACAGAAGAGCTGCAGGAAATG	276	62	Vankerckhoven et al. (2004)
		GACTGACGTCCAAGTTTCCAA			
Accessory colonization factor	ace	GAATTGAGCAAAAGTTCAATCG	1,108	60	McBride et al. (2009)
		GTCTGTCTTTTCACTTGTTTC A			
Endocarditis antigen	efaA	GCCAATTGGGACAGACCCTC	688	56	McBride et al. (2009)
		CGCCTTCTGTTCCTTCTTTGGC			
Tyrosine decarboxylase	tdc	ACATAGTCAACCATGTTGAA	924	60	Connil et al. (2002)
		CAAATGGAAGAAGAAGTAGG			
Ornithine decarboxylase	odc	CATCAAGGTGGACAATATTTCCG	1,446	56	Sánchez et al. (2019)
		CCGTTCAACAACTTGTTTGGCA			
Histidine decarboxylase	hdc	TTGACCGTATCTCAGTGAGTCCAT	367	62	Fernández et al. (2006)
		ACGGTCATACGAAACAATACCATC			

### 2.5 *In vivo* safety evaluation of LAB isolates

To evaluate the safety of the LAB isolates, BALB/c mice weighing 17–21 g (6 weeks of age) were obtained from Beijing Vital River Laboratory Animal Technology Co., Ltd., China, and acclimatized for 1 week before experiments. The animals were housed in an animal facility with free access to food and water and maintained in a 12 h light and 12 h dark cycle. The experimental protocol was approved by the Experimental Animal Ethics Committee of Harbin Veterinary Research Institute (HVRI), Chinese Academy of Agricultural Sciences with the license SYXK (Heilongjiang) 210903-01-GR.

The mice were divided into eight groups of five mice each. Seven experimental groups received oral doses of different LAB isolates at concentrations of 1 × 10^9^ CFU/100 μL, while the control group received 100 μL PBS. The general health parameters, including average daily gain (ADG), hematological analysis, and the organ index, were calculated after 21 days of continuous LAB supplementation to assess the safety of the LAB isolates. The mice were anesthetized and blood samples were collected from the retro-orbital sinus and then analyzed using the IDEXX ProCyte Dx Hematology Analyser (IDEX, United States). After blood sample collection, heart, lungs, liver, bilateral kidneys, spleen, PALN and AALN were harvested and weighed. The organ index was measured as follows: weight of organ/body weight of the mouse. Ileal tissue samples, 10 cm proximal to the cecum, were harvested and assessed for histopathological changes.

### 2.6 Anti-typhoidal activity of LAB isolates *in vivo*

The anti-infection ability of LAB isolates was evaluated in BALB/c mice, which were obtained and domesticated in the similar environment as above mentioned, with the *S. typhimurium* infection model (Vinderola et al., 2007). The Experimental Animal Ethics Committee of Harbin Veterinary Research Institute (HVRI), Chinese Academy of Agricultural Sciences, approved the experimental protocol with the license SYXK (Heilongjiang) 220718-01-GR. The mice were randomly assigned to four groups (*n* = 14 per group): two experimental groups, one for each tested isolate (LR6, LA6), and two control groups, one challenged with *S. typhimurium* (infected control, IC) and the other not challenged with the pathogen (normal control, NC). The experimental groups received oral doses of LR6 or LA6 isolates at a concentration of 1 × 10^9^ CFU/100 μL, while the control groups received 100 μL PBS. After 14 days of continuous LAB supplementation, the experimental groups and the infected control group were orally challenged with 100 μL of 1 × 10^8^ CFU/mL *S. typhimurium*. The mice were weighed throughout the experiment until day 10 post-infection. Three mice per group and assay were sacrificed at days 3 and 5 post-challenge and samples were obtained. Blood was collected from the heart of anaesthetized mice, and serum was separated by centrifugation at 3000 × *g* for 10 min. The spleen, liver, and ileum were dissected and. Rapidly divided into two parts: one stored in liquid nitrogen, and the other fixed in 4% paraformaldehyde. The survival of the remaining 8 mice in each group was monitored for another 10 days, and they were euthanized on day 11.

#### 2.6.1 Translocation assay

Under aseptic conditions, the spleen and liver were removed, weighed, and homogenized in tubes containing 2 mL of 0.1% peptone water. Serial 10-fold dilutions of the homogenates were prepared and 100 μL of each dilution was plated onto deoxycholate hydrogen sulfide lactose medium agar (Solarbio, China). The plates were incubated aerobically at 37°C for 24 h, and the number of colonies was counted.

#### 2.6.2 Assessment of the inflammatory cytokines

The level of serum tumor necrosis factor α (TNF-α) was quantified using ELISA kits (Neobioscience, China).

As described above, the liver, spleen, and ileum were removed from the mice. The tissues were homogenized, and total RNA was extracted from the homogenates using a Fast Pure Cell/Tissue Total RNA Isolation Kit (Vazyme, China). mRNA levels in the tissues were measured by quantitative real-time polymerase chain reaction (qRT-PCR) analysis using TB Green Premix Ex Taq (Tiangen, China) according to the instructions. The PCR primer sequences were as follows: GAPDH (5′-CGCGAGAAGATGACCCAGAT-3′, 5′-GCACTGTGTTGGCGTACAGG-3′); TNF-α (5′-CGTTGTAGCCAATGTCAAAGCC-3′, 5′-TGCCCAGATTCAGCAAAGTCCA-3′). The results were analyzed using QuantStudio5 (Applied Biosystems, United States) and β-actin was used as an internal standard. The final expression levels were obtained using the formula 2^−ΔΔCT^.

#### 2.6.3 Hematoxylin-eosin staining analysis

The liver, spleen, and ileum tissues were fixed in 4% paraformaldehyde for 72 h. The tissue blocks were dehydrated and embedded using alcohol, xylene, and paraffin, and then sectioned. The sections were dewaxed with xylene, rinsed with alcohol and distilled water five times, and stained with hematoxylin for 5 min. The sections were then washed with deionized water and stained with eosin for 1 min. The sections were dehydrated with ethanol, cleared with xylene, mounted on slides, and analyzed under a microscope.

### 2.7 Statistical analysis

All data were expressed as mean ± standard error of the mean (SEM). The data were analyzed using SPSS version 22 (IBM Corp., Armonk, NY, United States). One-way analysis of variance (ANOVA) was used to determine the significance among data sets. Statistical significance was set at: *, *p* < 0.05, **, *p* < 0.01, ***, *p* < 0.001.

## 3 Results

### 3.1 Isolation and identification of LAB isolates

Fifty-two pure bacterial colonies were obtained from SPF pigs with high antibody levels in this study. To identify the isolates, 16S rDNA gene sequencing analysis was performed. The results revealed that the 52 isolates included nine species, namely, *Lactobacillus animalis* (LA1–LA8), *Lactobacillus salivarius* (LS1–LS9), *Lactobacillus reuteri* (LR1–LR6), *Lactobacillus johnsonii* (LJ1), *Lactobacillus crispatus* (LC1, LC1), *Enterococcus Montessori* (EM1–EM4), *Enterococcus sakazakii* (EB1, opportunistic pathogen), *Pseudostaphylococcus* (PSC1, PSC2), *Enterococcus hirae* (EH1–EH4), and *Enterococcus faecalis* (EF1–EF15).

### 3.2 Probiotic characteristics of LAB isolates

#### 3.2.1 Antimicrobial activities of LAB isolates

The antimicrobial activity of the isolates was an important index for evaluating the prebiotic properties of the isolates. The 52 isolates were tested for their antimicrobial activity against three pathogens (*E coli, S. typhimurium and S. aureus*), using two commercial probiotics (*Lactobacillus acidophilus* (cLA) and *Lactobacillus plantarum* (cLP)) as control LAB strains. The strains and antimicrobial activities are listed in Table 2. Fourteen LAB isolates with high antimicrobial capacity (marked in red) were selected for the following experiments. LAB strains cLA and cLP were used as positive controls, and EB1 (opportunistic pathogen) as a negative control in the following experiments.

**Table 2:** Inhibition zones (mm) of all strains against selected microorganisms.

Isolates	Indicator pathogens		
	*E. coli* ATCC 8379	*S. typhimurium* ATCC 14028	*S. aureus* ATCC 25923
LA1	14.73 ± 0.08	16.2 ± 0.08	15.63 ± 0.42
LA2	14.7 ± 0.43	15.93 ± 0.31	18.03 ± 0.25
LA3	15.57 ± 1.21	15.27 ± 0.17	16.63 ± 0.34
LA4	15.23 ± 0.5	16.67 ± 0.39	13.7 ± 0.28
LA5	7	13.07 ± 0.21	11.53 ± 0.48
LA6	16.67 ± 0.39	16. 67 ± 0.39	17.77 ± 0.25
LA7	7	14.1 ± 0.22	15.53 ± 0.4
LA8	10.53 ± 0.48	9.93 ± 0.31	14.87 ± 0.4
LR1	15.13 ± 0.05	14.83 ± 0.34	15.37 ± 0.66
LR2	13.37 ± 1.23	11.67 ± 0.83	13.33 ± 0.94
LR3	11.53 ± 0.92	13.1 ± 0.43	19.1 ± 0.75
LR4	10.57 ± 0.45	16.3 ± 0.16	13.97 ± 0.4
LR5	15.67 ± 0.46	14.17 ± 0.05	15.97 ± 0.33
LR6	17.3 ± 0.65	14.97 ± 0.34	17.03 ± 0.17
LS1	13.93 ± 0.45	18.67 ± 0.39	17.7 ± 0.28
LS2	11.03 ± 0.62	16.57 ± 0.39	14.87 ± 0.57
LS3	14.3 ± 0.7	17.83 ± 0.45	18.27 ± 0.65
LS4	14.53 ± 0.87	17.87 ± 0.54	15 ± 0.57
LS5	14.93 ± 0.31	16.1 ± 0.43	18.6 ± 0.42
LS6	13 ± 0.36	15.97 ± 0.34	11.83 ± 0.45
LS7	14.33 ± 0.37	16.63 ± 0.17	13.77 ± 0.25
LS8	12.07 ± 0.80	15.77 ± 0.38	15.3 ± 0.14
LS9	16.67 ± 0.59	19.23 ± 0.05	20.43 ± 0.74
LJ1	15.77 ± 0.38	16.23 ± 0.05	15.17 ± 0.05
LC1	9.6 ± 0.37	9.27 ± 0.05	13.67 ± 0.39
LC2	10.87 ± 0.4	17 ± 0.51	14.67 ± 0.39
EB1	13.23 ± 0.25	13.07 ± 0.12	12.83 ± 0.25
EM1	7	7	7
EM2	9.67 ± 0.39	12.67 ± 0.31	13.17 ± 0.05
EM3	11.77 ± 0.25	13.93 ± 0.42	13.63 ± 0.34
EM4	7.33 ± 0.47	8.63 ± 0.34	7
EH1	9.93 ± 0.31	12.13 ± 0.05	11.27 ± 0.17
EH2	10.03 ± 0.25	11.43 ± 0.17	10.87 ± 0.4
EH3	10.27 ± 0.05	11.93 ± 0.31	10.3 ± 0.75
EH4	10.93 ± 0.31	48.03 ± 51.6	10.93 ± 0.31
PSC1	7	7	7
PSC2	10.63 ± 0.34	11.77 ± 0.25	11.93 ± 0.31
EF1	10.73 ± 0.56	9.8 ± 0.29	10.63 ± 0.34
EF2	7	11.77 ± 0.25	11.13 ± 0.05
EF3	10.8 ± 0.24	10.8 ± 0.29	14.27 ± 0.65
EF4	11.03 ± 0.41	10.3 ± 0.16	11.9 ± 0.92
EF5	7	9.83 ± 0.29	7
EF6	7	7	7
EF7	7	9.83 ± 0.29	7
EF8	7	10.73 ± 0.56	11.27 ± 0.17
EF9	7	11.07 ± 0.42	11.15 ± 0.05
EF10	7	10.93 ± 0.31	7
EF11	7	11.17 ± 0.05	7
EF12	7	10.83 ± 0.38	7
EF13	7	10.4 ± 0.14	7
EF14	7	7	7
EF15	7	7	7
cLA	14.34 ± 0.74	14.54 ± 0.91	15.83 ± 0.42
cLP	13.77 ± 0.65	13.53 ± 0.45	13.77 ± 0.31
MRS	7	7	7

Values are means ± standard deviations of triplicates (mm), agar perforator with a diameter of 7 mm, the 14 LAB isolates with high antimicrobial capacity are marked in red.

#### 3.2.2 Autoaggregation and coaggregation

**Figure 1:**
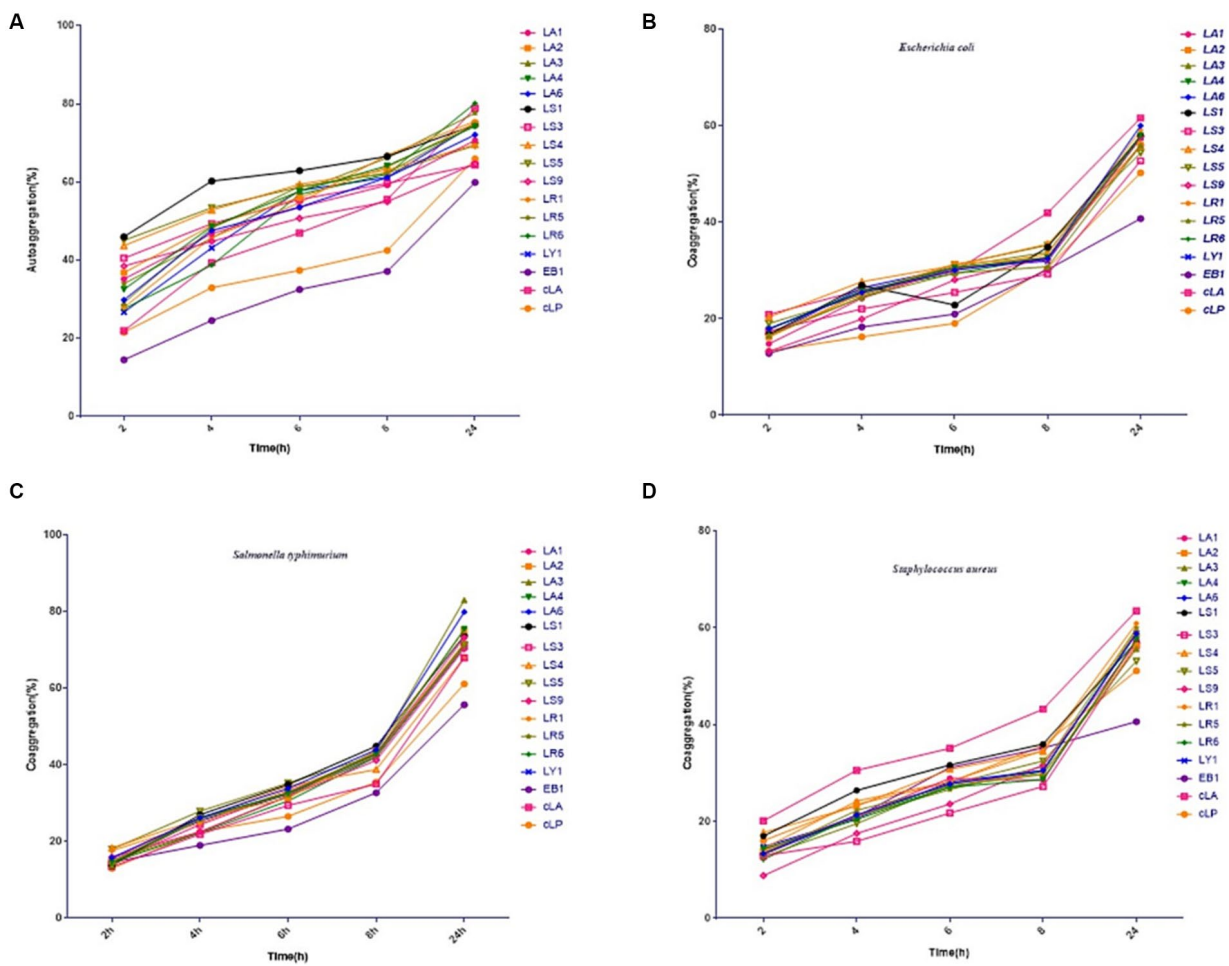
Percentages of autoaggregation **(A)** and coaggregation with enteropathogenic *E. coli* **(B)**, *Salmonella typhimurium* **(C)**, and *Staphylococcus aureus* **(D)** in the 14 LAB isolates with better antimicrobial capacity, along with EB1 and two commercial strains (cLA, cLP). Data represent means ± standard deviations.

The 14 LAB isolates were assessed for their autoaggregation and coaggregation abilities. As shown in Figures 1A–C, all strains exhibited autoaggregation, which progressively and significantly increased over time. After 24 h of incubation, similar autoaggregation percentages ranging from 64 to 80% were observed for all isolates except for the EB1 (59% autoaggregation) (Figure 1A). Compared to EB1, the 14 LAB isolates and two commercial strains had better coaggregation with all tested pathogens (Figures 1B–D). The coaggregation of the 14 isolates and two commercial strains with *S. typhimurium* was higher than that with *E. coli* and *S. aureus* (Figures 1B–D). Figure 1 also showed that most of the 14 LAB isolates had higher autoaggregation and coaggregation abilities than the two commercial strains and EB1 (opportunistic pathogen).

#### 3.2.3 Acid and bile salt tolerance of LAB isolates

**Figure 2:**
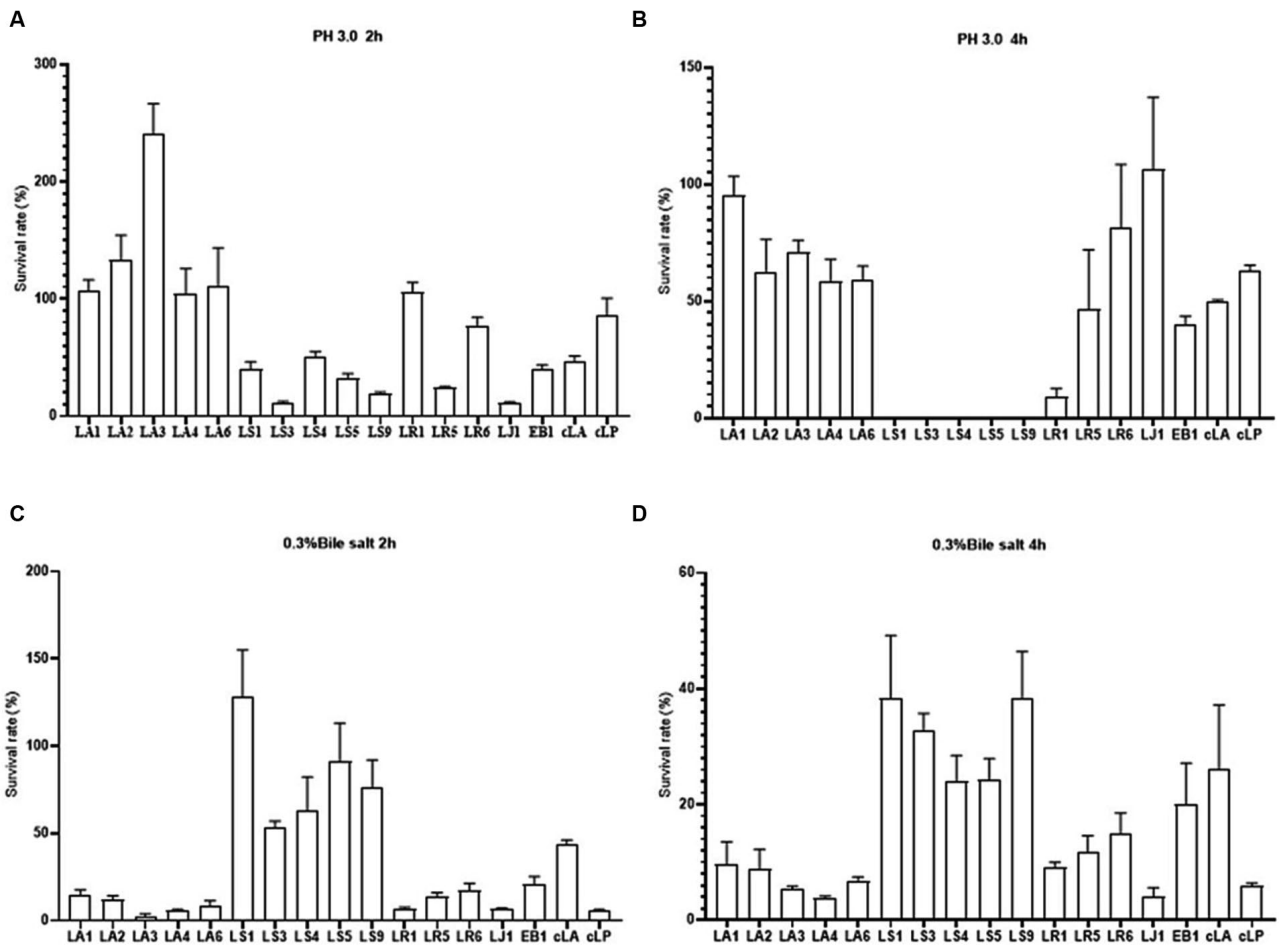
Survival rates of the 14 LAB isolates with better antimicrobial capacity, along with EB1 and two commercial strains in acid for 2 h **(A)** and 4 h **(B)** and in bile salt for 2 h **(C)** and 4 h **(D)**.

The tolerance of gastrointestinal tract (GIT) conditions is an important criterion for selecting potential probiotics. Hence, we assessed the survival of the 14 LAB isolates under acid and bile salt conditions. As shown in Figures 2A,C, all strains tested had the ability to survive for 2 h under low pH or 0.3% bile salt condition, with different survival rates among strains. LS had a weak ability to survive at low pH, and nearly all LS died after 4 h of incubation at pH 3.0 (Figures 2A,B). However, LS survived better at 0.3% bile salt (Figures 2C,D). These results may be due to the fact that LAB mainly colonize the gut, making them more tolerant of bile salt and less tolerant of the low pH environment of the stomach. In summary, all the 14 strains have the potential to colonize the gut.

#### 3.2.4 Adhesion properties of LAB isolates

**Figure 3:**
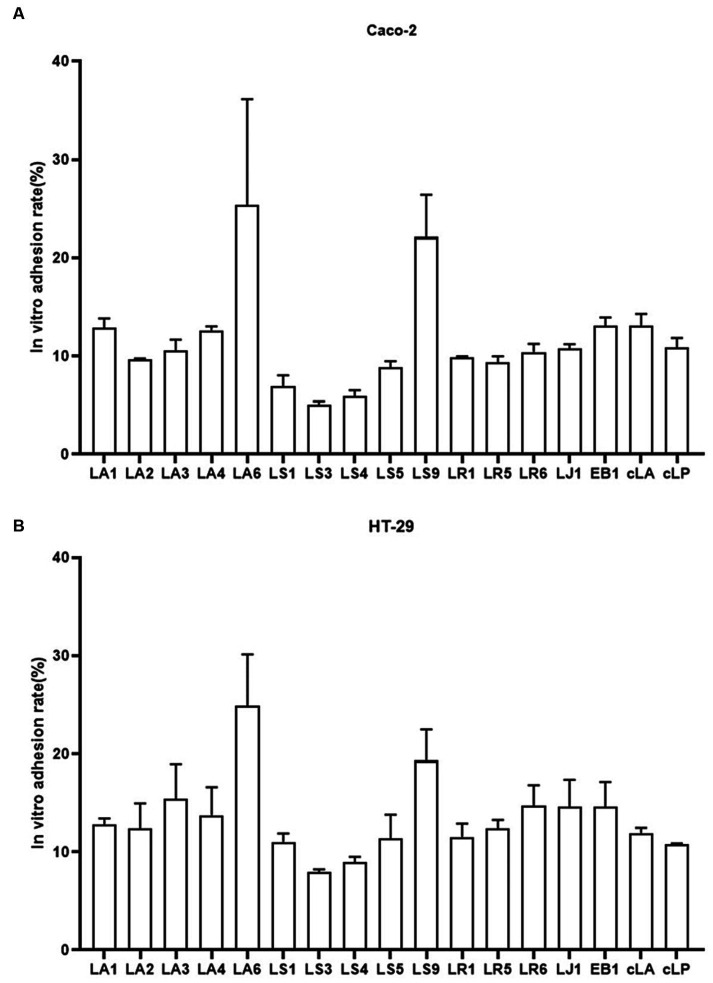
Adhesion of the 14 LAB isolates with better antimicrobial capacity, along with EB1 and two commercial strains to HT-29 **(A)** and Caco-2 **(B)** cells. Data represent means ± standard deviations.

Probiotics are live microorganisms that confer health benefits when administered in adequate amounts. To be classified as probiotics, LAB must reach the intestine through the stomach and duodenum and attach to intestinal epithelial cells. Therefore, we examined the adhesion abilities of the 14 LAB isolates to HT-29, and Caco-2 cells (Figures 3A,B). We found that, exception for LS9, LA and LR adhered better to HT-29 and Caco-2 cells than LS, while LA6 and LS9 showed stronger adhesion properties than the other strains (Figures 3A,B).

### 3.3 *In vitro* safety evaluation of LAB isolates

Safety is crucial for the development of probiotic isolates in clinical applications. A safety prerequisite for selecting a probiotic strain is an absence of hemolytic activity (FAO/WHO, 2002). Therefore, we analyzed the hemolytic activity of the 14 LAB isolated strains using blood agar plates and found that none of the 14 LAB isolated strains showed hemolytic activity (Supplementary Figures S1A–H).

Although of LAB is generally considered safe, some studies have shown that certain members of the LAB genus can cause health problems such as bacteremia, endocarditis, and peritonitis (Colautti et al., 2022). Therefore, identifying virulence genes in the isolates is a crucial indicator of the safety of probiotics. We tested the 14 LAB strains for the presence of 10 virulence genes including *asa1, gelE, cylA, esp., hyl, ace, efaA, tdc, odc* and *hdc*. The results showed that only strains LS1 and LS9 had the virulence gene *efaA*, while the other strains had none of the ten virulence genes (Supplementary Figure S1K).

Next, the antibiotic susceptibility pattern of the 14 LAB isolates against different antibiotics (16 antibiotics from 10 classes) was determined by disc diffusion assay. Data for all strains tested can be found in Table 3. Almost all the strains tested, including two commercial probiotics, exhibited resistance to tetracycline antibiotics, quinolone antibiotics, peptide antibiotics, sulfonamide antibiotics, aminoglycoside antibiotics and penciclovir among β-lactam antibiotics. All 14 isolates except LS were susceptible to macrolide antibiotics, lincomycin antibiotics, chloramphenicol antibiotics, nitrofuran antibiotics and penicillin among β-lactam antibiotics. LS was only susceptible to chloramphenicol antibiotics and penicillin among β-lactam antibiotics.

**Table 3:** Antibiotic susceptibility profiles of the 17 strains.

Antibiotic category	Name of antibiotic	LA1	LA2	LA3	LA4	LA6	LS1	LS3	LS4	LS5	LS9	LR1	LR5	LR6	LJ1	EB1	cLA	cLP
Macrolide	Erythromycin	S	S	S	S	S	R	R	R	R	R	S	S	S	S	S	I	S
	Clarithromycin	S	S	S	S	S	R	R	I	I	R	S	S	S	S	S	R	S
Lincomycin	Clindamycin	S	S	S	S	S	R	R	R	R	R	S	S	S	S	S	S	S
Chloramphenicol	Chloramphenicol	S	S	S	S	S	S	S	S	S	S	S	S	S	S	S	S	S
Nitrofuran	Nitrofurantoin	S	S	S	S	S	I	I	I	I	S	S	S	S	S	S	I	S
Beta-lactam	Penicillin	S	S	S	S	S	S	S	S	S	S	S	S	S	S	S	R	R
	Oxacillin	R	I	R	R	R	R	R	R	R	R	R	I	I	R	R	I	R
Tetracycline	Tetracycline	R	R	R	R	R	R	R	R	R	R	R	R	R	R	R	I	I
	Minocycline	R	S	R	I	R	R	I	I	I	I	R	I	I	R	I	I	R
Quinolone a	Norfloxacin	R	R	R	R	R	R	R	R	R	R	R	R	R	R	R	R	R
	Ciprofloxacin	R	R	R	R	R	I	R	I	I	I	R	R	R	R	R	R	R
	Ofloxacin	R	R	R	R	R	R	R	R	R	R	R	R	R	R	R	R	R
Polypeptide	Vancomycin	R	R	R	R	R	R	R	R	R	R	R	R	R	R	R	R	R
	Polymyxin B	R	R	R	R	R	R	R	R	R	R	R	R	R	R	R	R	R
Sulfonamides	Sulfamethoxazole	R	R	R	R	R	R	R	R	R	R	R	R	R	R	R	R	S
Aminoglycoside	Spectinomycin	R	R	R	R	R	I	R	R	R	R	R	R	R	R	R	I	R

“S” indicates sensitive, “R” indicates insensitive, and “I” indicates intermediate between sensitive and insensitive.

### 3.4 *In vivo* safety assessment of LAB isolates

**Figure 4:**
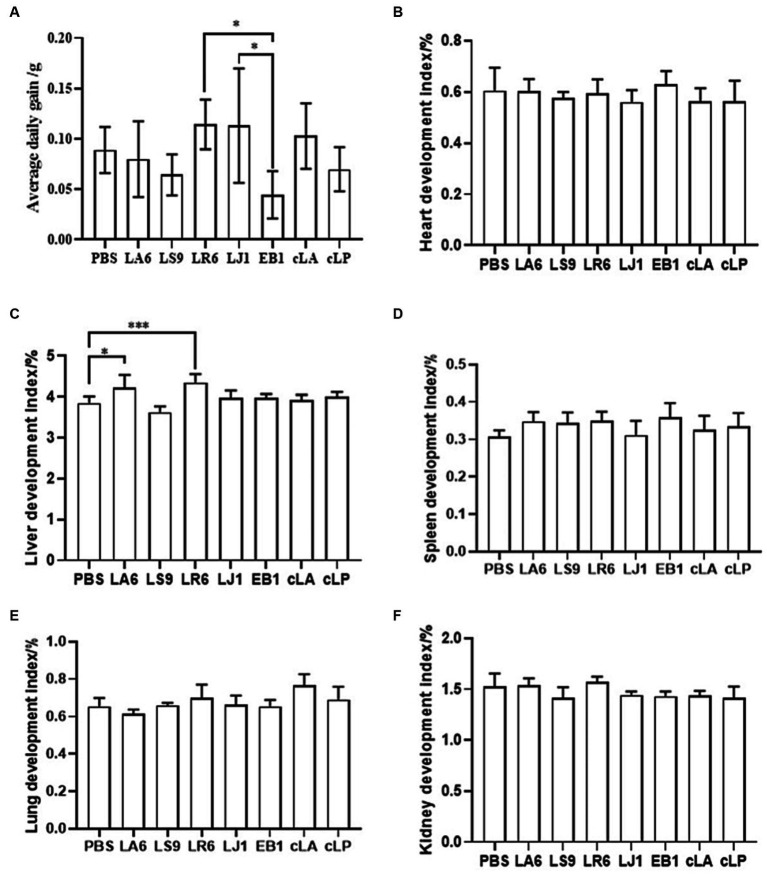
Effect of LAB supplementation on body weight gain and organ indices of experimental mice. **(A)** ADG of mice gavaged with selected strains. **(B–F)** The heart **(B)**, liver **(C)**, spleen **(D)**, lung **(E)**, and kidney **(F)** indices in the experimental groups. Data represent means ± standard deviations. *p < 0.05, ***p < 0.001.

**Figure 5:**
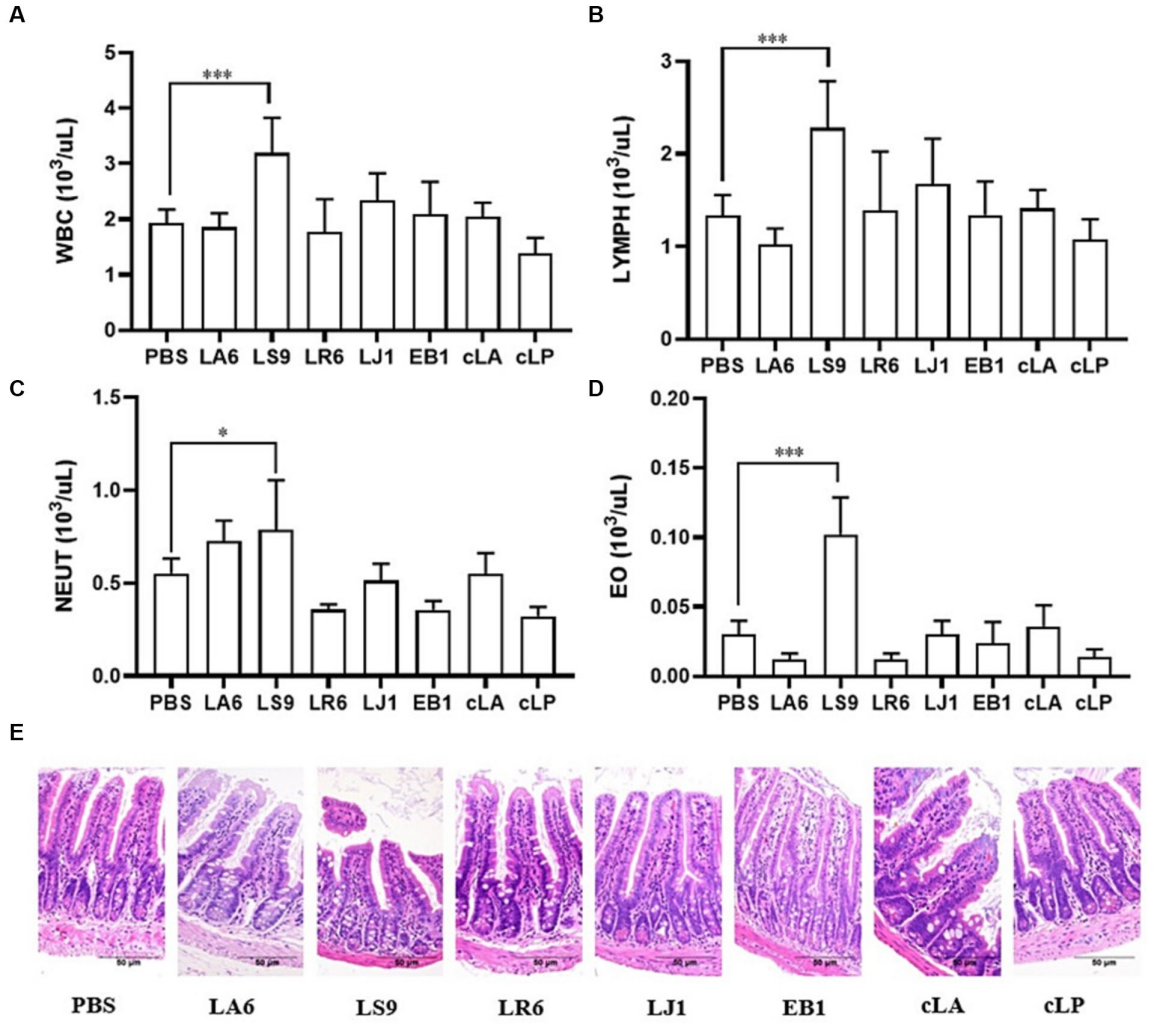
Effects of *strains* supplementation on blood immune cells and the ileum of experimental mice. **(A–D)** The numbers of leukocytes, lymphocytes **(B)**, neutrophils **(C)**, and eosinophils **(D)** in the blood of mice. **(E)** HE staining of ileal tissue sections to evaluate the general morphological changes. Data represent means ± standard deviations. *p < 0.05, ***p < 0.001.

Ensuring the safety of LAB isolates *in vivo* is a crucial factor for clinical application. Four LAB strains with high probiotic potential were selected from 14 LAB strains of four different species (LA, LR, LJ and LS) based on the results of previous experiments. They were LA6, LR6, LJ1 and LS9 (which showed the best performance despite containing a virulence gene). We conducted a preliminary assessment of the safety of LA6, LR6, LJ1, LS9 in mice. Two commercial probiotics (cLA and cLP) were used as positive control and strain EB1 was used as negative control. The LR6, LJ1 and cLA groups showed an increase in the average daily gain (ADG), while the LS9 and EB1 groups showed a decrease in ADG compared to the PBS group; however, these differences were not statistically significant (*p* > 0.05) (Figure 4A). Moreover, the ADG values in the EB1 group were significantly lower than those in the LR6 and LJ1 groups (*p* < 0.05) (Figure 4A). In addition, apart from a significant increase in liver development index in groups LA6 and LR6 compared to the PBS group (Figure 4C), no significant differences in other organ indices among the different groups were observed (Figures 4B–F). Blood cell populations were analyzed by IDEXX ProCyte Dx Hematology Analyser (IDEX, United States). As shown in Figures 5A–D, the strain LS9 led to a significant increase in the number of lymphocytes (*p* < 0.001), neutrophils (*p* < 0.05) and eosinophils (*p* < 0.001) in the blood of mice. Meanwhile, the effects of these strains on the intestinal tract were examined by analyzing H&E-stained ileal sections from mice in different groups. Only the mice in the LS9 group exhibited a marked thinning of the ileal wall, as well as villous injury, shortening, and atrophy. No abnormalities were observed in the ileal wall or villi of the other groups (Figure 5E).

### 3.5 Lab isolates attenuate *Salmonella typhimurium* infection in mice

**Figure 6:**
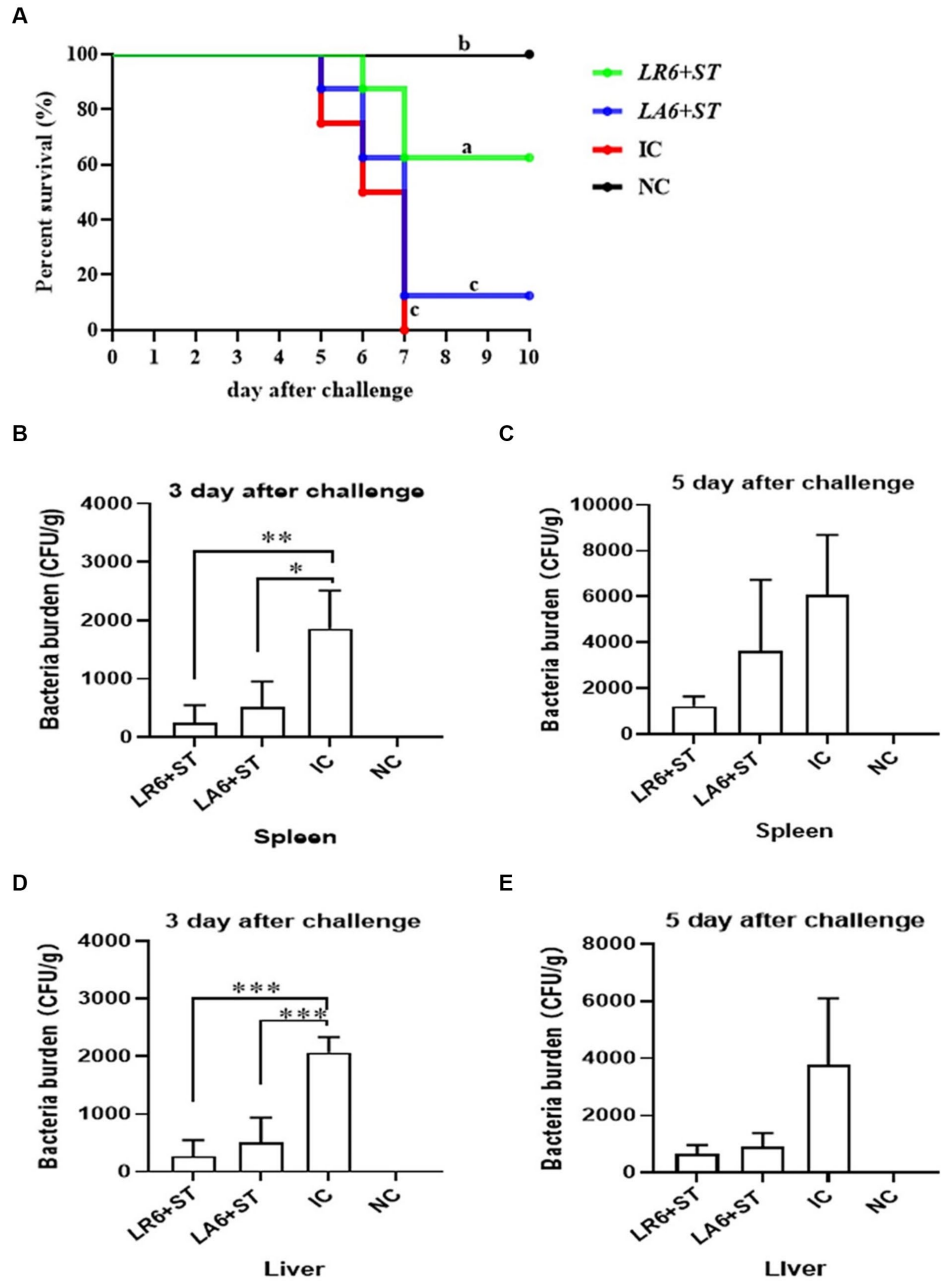
Effects of *LR6* and *LA6* on mortality and bacterial translocation in mice infected with *S. typhimurium* (ATCC14028). **(A)** The survival rate of mice in each group after infection. **(B–E)** Day 3 and day 5 after challenge, the number of bacteria in the spleen **(B,C)** and liver **(D,E)**. Data represent means ± standard deviations. *p < 0.05, **p < 0.01, ***p < 0.001.

To further determine the antimicrobial abilities of LAB isolates, which exhibited better antimicrobial activity *in vitro*, an *in vivo* experiment was conducted. Two LAB isolates, LR6 and LA6, the best overall performers of LR and LA respectively, were chosen to evaluate their resistance to bacterial infection using the *S. typhimurium* infection model in mice. As shown in Figure 6A, all mice in the IC group succumbed to the *S. typhimurium* challenge within 7 days, whereas the survival rate of the LR6 treatment group was significantly higher (62.5%) than that of the LA6 and IC groups (12.5 and 0%, respectively) (*p* < 0.01). The bacterial loads in the liver and spleen of mice in the LR6 and LA6 treatment groups were significantly lower than those in the IC group on day 3 after challenge (Figures 6B,D), the bacterial loads in the liver and spleen of mice in the LR6 and LA6 treatment groups were still lower than those in the IC group on day 5 after challenge, but the difference was not significant (*p* > 0.05) (Figures 6C,E). These results suggest that pre-treatment with LR6 and LA6 reduced the spread of *S. typhimurium* to distal organs. The LR6 treatment group demonstrated a superior ability to decrease the bacterial load compared with the LA6 treatment group.

**Figure 7:**
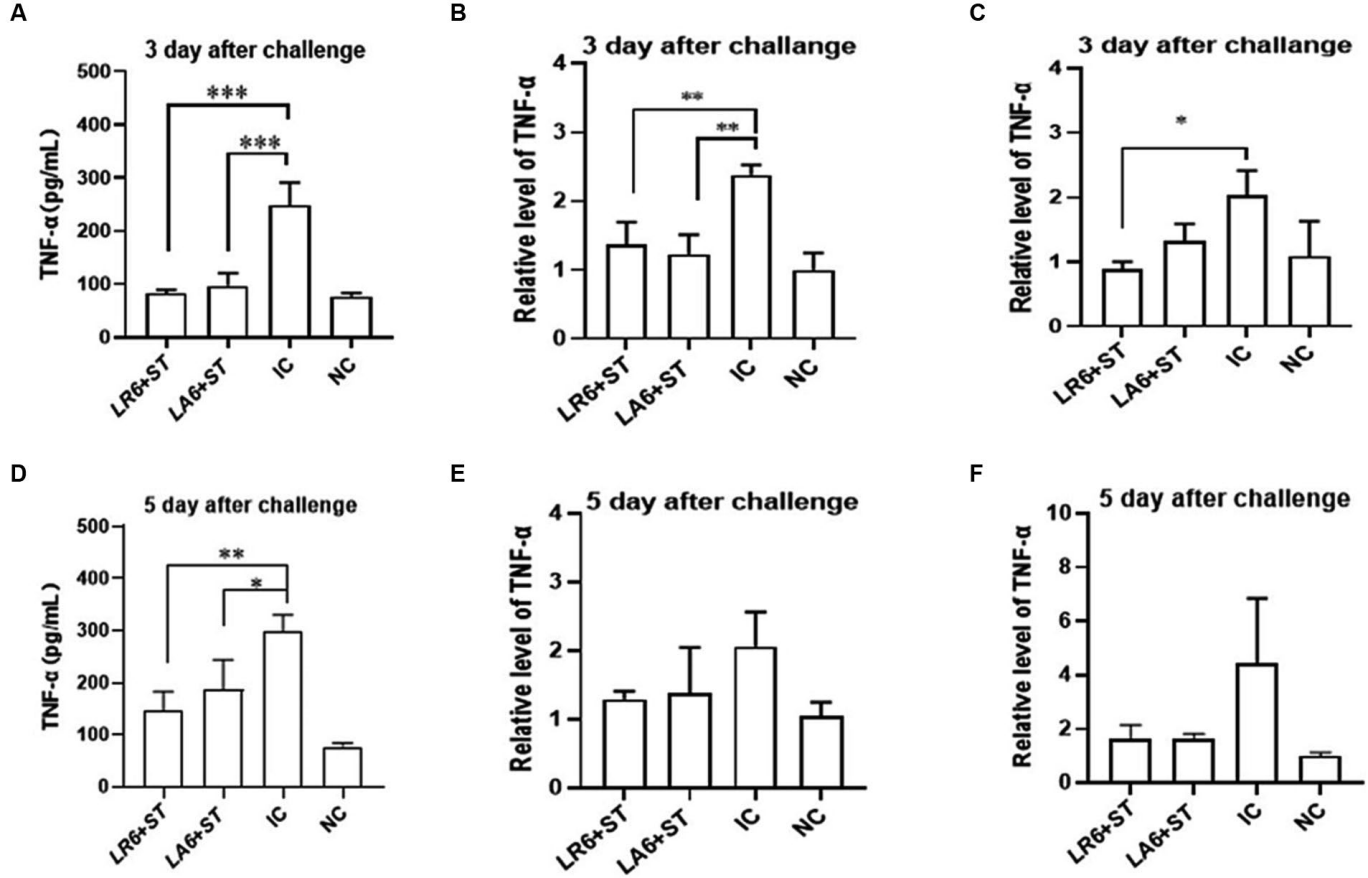
Effect of *LR6* and *LA6* on inflammatory cytokines in mice infected with *S. typhimurium* (ATCC 14028). **(A,D)** TNF-α levels in mice serum at day 3 and day 5 after challenge. **(B,E)** Expression of TNF-α in spleen on day 3 and day 5 after challenge. **(C,F)** Expression of TNF-α in liver on day 3 and day 5 after challenge. Data represent means ± standard deviations. *, *p* < 0.05; **, *p* < 0.01; ***, *p* < 0.001.

Furthermore, the concentration of tumor necrosis factor α (TNF-α) in the blood and the mRNA levels of TNF-α in the spleen and liver were quantified using ELISA and qPCR, respectively, on days 3 and 5 after challenge. As shown in Figures 7A–F, the serum and the mRNA levels of TNF-α in the spleen and liver of mice in the IC group were significantly higher than those in the NC group on days 3 and 5 after challenge. Pretreatment with either LR6 or LA6 significantly reduced the serum levels of TNF-α, which were induced by *S. typhimurium* infection on day 5 after challenge (*p* < 0.001) (Figure 7A). This treatment also notably decreased the mRNA level of TNF-α in the spleen (*p* < 0.01) (Figure 7B). Interestingly, only the pretreatment with LR6 significantly reduced the mRNA level of TNF-α (*p* < 0.05). Although pretreatment with LA6 also reduced the mRNA level of TNF-α in the liver, the difference was not statistically significant (*p* > 0.05) (Figure 7C). The serum levels of TNF-α in mice pretreated with LR6 and LA6 were also found to be lower than those of the IC group on day 5 after challenge (*p* < 0.01, *p* < 0.05) (Figure 7D). Additionally, the mRNA levels of TNF-α in the spleens and livers of these pretreated mice were also lower than those in the IC group, but the difference was not statistically significant (*p* > 0.05) (Figures 7E,F).

**Figure 8:**
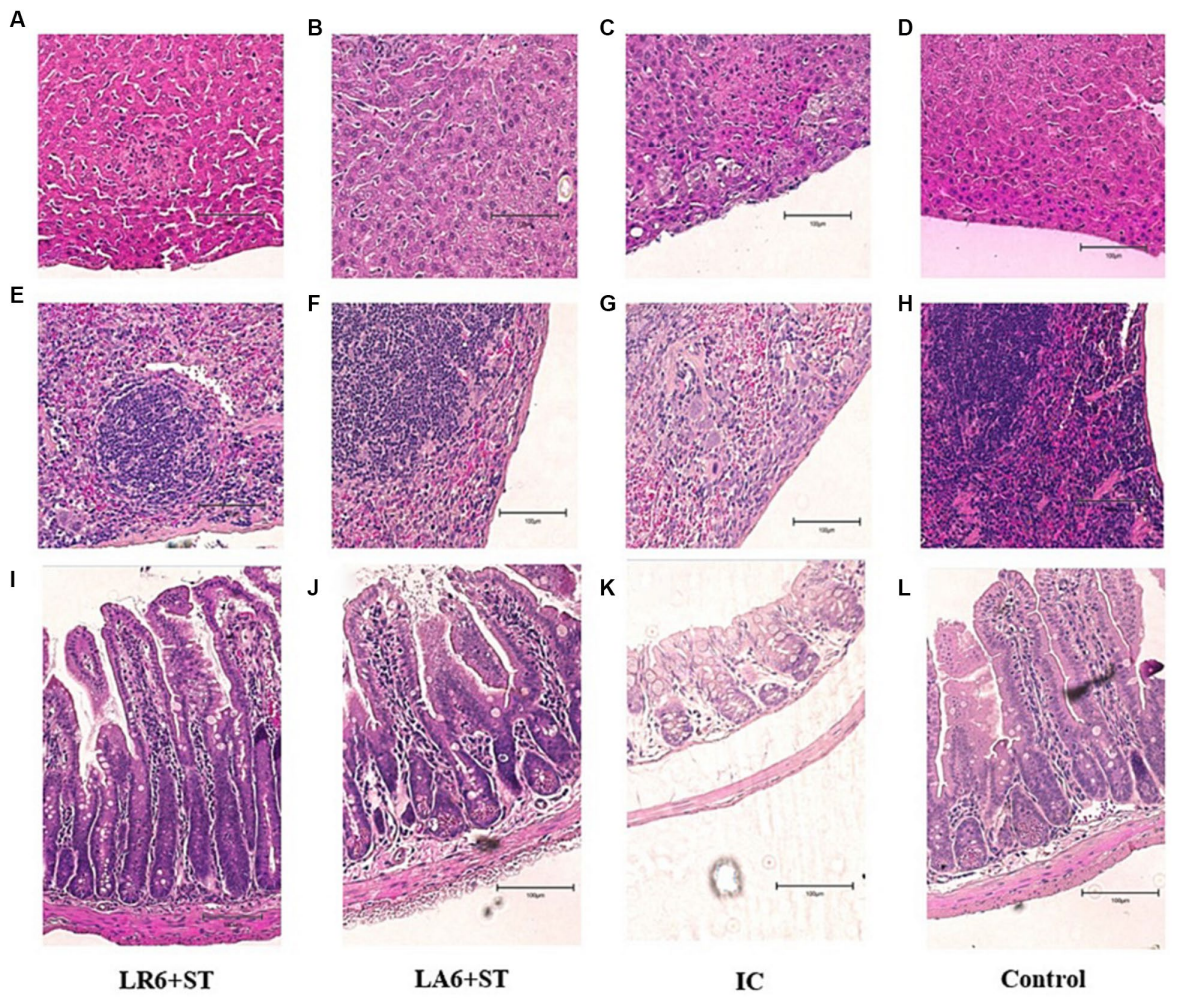
Effect of *LR6* and *LA6* on alleviating the liver, spleen, and ileum injury caused by *S. typhimurium* (ATCC 14028) infection in mice. Histological morphology of the **(A–D)** liver, **(E–H)** spleen, and **(I–L)** ileum (H&E staining). **(A,E,I)** Mice fed LR6 and challenged with *S. typhimurium*, **(B,F,J)** mice fed LA6 and challenged with *S. typhimurium*, **(C,G,K)** control mice challenged with *S. typhimurium*, **(D,H,L)** control mice fed PBS.

As shown in the H&E-stained histopathological images (Figures 8A–L), the infection of *S. typhimurium* resulted in marked lesions in the liver, spleen and ileum of mice, including multiple inflammatory granulomatous nodules in the liver parenchyma with extensive hepatocellular necrosis, a marked reduction in lymphocytes in the white pulp of the spleen, focal inflammatory cell infiltration in the red pulp (predominantly neutrophils), and massive infiltration of neutrophils and monocytes in the lamina propria and submucosal layer of the ileum, with marked submucosal oedema and hemorrhage. Pretreatment with LR6 and LA6 reduced the extent of lesions in the liver, spleen and ileum, with the LR6 treatment group showing superior outcomes.

## 4 Discussion

It is well known that LAB are considered beneficial microorganisms that modulate the intestinal function, immunity, and microbiota of the host, thus enhancing the overall health of the host (Wang et al., 2019). Several studies have shown that the gut microbiota composition and its interaction with the immune system influence the development and function of humoral immunity and vaccine efficacy (Zhang et al., 2018; Ling et al., 2020; Ran et al., 2021). A close association between LAB abundance in pig intestinal microbiota and higher antibody titers, reduced pathogenic damage, and faster PRRSV clearance was found in our previous study. The group with higher antibody titer had a significantly greater LAB proportion (18.95%) than the group with lower antibody titer (4.81%) (Zhang et al., 2021).

Based on our previous findings, we hypothesized that LAB with strong immunoregulatory properties could be isolated from pigs with good immune responses and abundant LAB, based on our previous findings. However, systematic evaluation of the isolated LAB was required to ensure their efficacy and safety. Therefore, this study aims to analyze the potential probiotic characteristics and safety of the LAB isolated from pigs with high antibody titers, and to test their probiotic effects in protecting mice against *S. typhimurium* infection for animal feed additives. To our best knowledge, this is the first report on the probiotic characteristics of the LAB strains isolated from pigs with high antibody titers and abundant LAB.

In this study, 52 strains belonging to nine species were identified: *Lactobacillus animalis*, *Lactobacillus sali*var*ius*, *Lactobacillus reuteri*, *Lactobacillus johnsonii*, *Lactobacillus crispatus*, Enterococcus Montessori, Enterococcus sakazakii, Pseudostaphylococcus, *Enterococcus hirae*, and *Enterococcus faecalis*. These results were in line with previous studies that suggested that LAB strains are prevalent in pigs (König et al., 2021; Zhong et al., 2022).

Increasing evidence suggests that certain probiotic strains can prevent infection by pathogenic intestinal microbes (Ghattargi et al., 2018; Saboori et al., 2022). Therefore, we tested the antibacterial activities of the 52 isolates and two commercial probiotics (*Lactobacillus acidophilus* and *Lactobacillus plantarum*) against various bacterial pathogens. Fourteen strains with strong antibacterial ability were selected, along with *Enterococcus sakazakii* (EB1, opportunistic pathogen) and two commercial probiotics, for further experiments. *L. animalis* (LA), *L. salivarius* (LS), or *L. reuteri* (LR) were the most common LAB isolates with strong antibacterial activity. The antimicrobial activity of LAB strains depended on their production of organic acids, carbon dioxide, hydrogen peroxide, diacetyl, fatty acids, bacteriocins, and bacteriocin-like substances, among others (Bojanic Rasovic et al., 2017).

Autoaggregation and coaggregation can prevent pathogen colonization of the intestinal surface by probiotics (Sharma et al., 2019). Bacteria often exist in consortia, adhering either to surfaces, non-bacterial cells, or other bacteria. Autoaggregation is the bacterium-bacterium adhesion of genetically identical strains, which enables microorganisms of the same species to aggregate and adhere to the intestinal mucosa (Lukic et al., 2014). Coaggregation is the inter-strain adherence of genetically distinct strains, of the same or different species, which facilitates the intercellular adhesion between different strains and their interaction with pathogens, enhancing host defense against infection (Zhang et al., 2013a). We observed that LAB species isolated from the same SPF pig had similar autoaggregation and coaggregation abilities, but different species had different abilities. LA and LS had significantly higher autoaggregation and coaggregation abilities EB1 and the commercial probiotic cLP. This may result from complex interactions between bacterial surface molecules (such as proteins) and secreted factors (Kuppusamy et al., 2020).

Bile salts in the duodenum and acidic conditions in the stomach are major challenges for LAB survival in the host gastrointestinal tract (Hsu et al., 2018). We found that LA and LR tolerated acidic environments better than LS, which was more tolerant of bile salt. These differences in resistance to acid environments and bile salts may be attributed to the expression of specific proteins in LAB cells (Hamon et al., 2011) and some bacterial proteins (Yu et al., 2013).

Adhere to the host gut is a key factor for probiotics to promote health (Iñiguez-Palomares et al., 2011). Adherence not only enables probiotics to survive longer in the gastrointestinal tract and enhances the interaction between bacteria and the host but also helps them overcome the effects of gastric motility (Jose et al., 2015). Thus, adherence to the mucosal surface and epithelial cells is an important property of probiotics. We used two intestinal cell lines, HT-29, and Caco-2, to assessed the adhesion characteristics of the isolates. LA and LR adhered better to HT-29 and Caco-2 than LS (except for LS9), and LA6 and LS9 had stronger adhesion than the other strains. This may result from specific cell surface molecules of each strain that affect their adherence to gut cells. These molecules may mediate their attachment to the intestinal mucosa and modulate the immune system.

LAB are generally considered non-pathogenic for humans (Aaron et al., 2017), but virulence genes from LAB isolates may transfer to other pathogenic bacteria (Al-Talib et al., 2015). In our study, none of the isolated caused hemolysis of sheep blood erythrocytes and only LS1 and LS9 carried virulence genes. Thus, except for LS1, LS9, and the opportunistic pathogen EB1, the other isolates were safe probiotic candidates. Antibiotic resistance is an important factor for LAB safety assessment (Guo et al., 2016). Antibiotic resistance may help beneficial microorganisms survive in the gastrointestinal tract (Zommiti et al., 2017). We found that all isolates except LS were susceptible to macrolide antibiotics, lincomycin antibiotics, chloramphenicol antibiotics, nitrofuran antibiotics and penicillin among β-lactam antibiotics but resistant to tetracycline antibiotics, quinolone antibiotics, peptide antibiotics, sulfonamide antibiotics, aminoglycoside antibiotics and penciclovir among β-lactam antibiotics. LS was only susceptible to chloramphenicol antibiotics and penicillin among β-lactam antibiotics. This result may reflect the natural resistance of LAB and the extensive use of antibiotics in pig production (Li et al., 2020; Wang M. et al., 2020).

Ensuring the safety of LAB isolates *in vivo* is a crucial factor for clinical application. Four LAB strains with high probiotic potential were selected from 14 LAB strains of four different species (LA, LR, LJ and LS), based on the results of above experiments. They were LA6, LR6, LJ1 and LS9 (which exhibited best performance despite containing a virulence gene). We then assessed the *in vivo* safety of LA6, LR6, LJ1, LS9 (with a virulence gene), EB1 (opportunistic pathogen) and two commercial probiotics (cLA, cLP) in mice. We monitored body weight loss, immune cell counts in the blood, and ileal tissue damage after 3 weeks of feeding with strains. We found that LR6 and LA6 supplementation increased growth performance and the liver index. These results showed that all the LAB isolates except LS9 were safe for mice, which was in agreement with previous studies (Aaron et al., 2017; Campagne et al., 2020). These results also suggested that virulence genes detection in the LAB isolates was necessary.

Published studies have mainly focused on probiotic metabolism and neglected their immune response (Yang et al., 2017; Gokhale and Bhaduri, 2019). Most studies on probiotics effects on pathogens were *in vitro* (Gao et al., 2022; Kiššová et al., 2022) and have commonly used intestinal pathogens (Wu et al., 2016; Wang W. et al., 2020). *S. typhimurium*, a pathogen that causes systemic disease in mice (Ballesté-Delpierre et al., 2017; Benoit et al., 2019), reflects LAB effects on systemic immune response better than intestinal pathogens. We chose two LAB isolates, LR6 and LA6, the best overall performers of LR and LA respectively, to evaluate their resistance to bacterial infection using the *S. typhimurium* infection model in mice. We found that treatment with LR6 and LA6 decreased serum TNF-α levels, and reduced the increased expression of the inflammatory factor TNF-α in the liver and spleen caused by *S. typhimurium* infection. Treatment with LR6 and LA6 also reduced *S. typhimurium* translocation to the liver and spleen, and alleviated liver, spleen and ileum damage caused by *S. typhimurium* infection. Moreover, the LR6 treatment group suppressed inflammatory response, decreased bacterial translocation and alleviated liver, spleen and ileum injury more than LA6 treatment group, which may explain the low mortality in the L6 treatment group. These results were in line with previous studies showing that LAB strains modulate immunity and benefit mice challenged with *S. typhimurium*, but their effects are strain-specific (Hirano et al., 2017; Abatemarco Júnior et al., 2018).

Taken together, we obtained a series of LAB isolates from pigs with high antibody titers and abundant LAB. These LAB isolates resisted bile salts and acids, and had strong capabilities of autoaggregation, coaggregation, antimicrobial activity and adhesion. We assessed their antibiotic resistance profiles, and verified their safety and potential as antimicrobial and probiotic agents *in vitro* and *in vivo*. These results showed that these LAB isolates, especially LR6, possessed remarkable probiotic properties, and warranted further investigation as potential feed additives.

## Data availability statement

The original contributions presented in the study are included in the article/Supplementary material, further inquiries can be directed to the corresponding authors.

## Ethics statement

The animal study was approved by Harbin Veterinary Research Institutional Animal Care Committee. The study was conducted in accordance with the local legislation and institutional requirements.

## Author contributions

WM: Conceptualization, Data curation, Formal analysis, Investigation, Methodology, Writing – original draft, Writing – review & editing. WZ: Conceptualization, Data curation, Formal analysis, Investigation, Methodology, Project administration, Writing – review & editing. XW: Conceptualization, Formal analysis, Writing – review & editing. YP: Conceptualization, Methodology, Writing – review & editing. MW: Methodology, Writing – review & editing. YX: Conceptualization, Writing – review & editing. JG: Methodology, Writing – review & editing. HCu: Methodology, Writing – review & editing. CL: Methodology, Writing – review & editing. HCh: Conceptualization, Funding acquisition, Writing – review & editing. HZ: Methodology, Project administration, Supervision, Writing – review & editing. CX: Methodology, Writing – review & editing, Funding acquisition, Supervision. YW: Conceptualization, Funding acquisition, Methodology, Supervision, Visualization, Writing – review & editing, Data curation, Project administration, Resources, Validation, Writing – original draft.

## References

[ref1] Aaron J. G. Sobieszczyk M. E. Weiner S. D. Whittier S. Lowy F. D. (2017). *Lactobacillus rhamnosus* endocarditis after upper endoscopy. Open Forum Infect. Dis. 4:ofx085. doi: 10.1093/ofid/ofx085, PMID: 28695143 PMC5499731

[ref2] Abatemarco Júnior M. Sandes S. H. C. Ricci M. F. Arantes R. M. E. Nunes Á. C. Nicoli J. R.. (2018). Protective effect of *Lactobacillus diolivorans* 1Z, isolated from Brazilian kefir, against *Salmonella enterica* Serovar typhimurium in experimental murine models. Front. Microbiol. 9:2856. doi: 10.3389/fmicb.2018.02856, PMID: 30564201 PMC6288297

[ref3] Afolayan A. O. Ayeni F. A. Ruppitsch W. (2017). Antagonistic and quantitative assessment of indigenous lactic acid Bacteria in different varieties of Ogi against gastrointestinal pathogens. Pan Afr. Med. J. 27:22. doi: 10.11604/pamj.2017.27.22.9707, PMID: 28748023 PMC5511708

[ref4] Alard J. Peucelle V. Boutillier D. Breton J. Kuylle S. Pot B.. (2018). New probiotic strains for inflammatory bowel disease management identified by combining in vitro and in vivo approaches. Benef. Microbes 9, 317–331. doi: 10.3920/bm2017.0097, PMID: 29488412

[ref5] Ali A. Imran M. Sial S. Khan A. (2022). Effective antibiotic dosing in the presence of resistant strains. PLoS One 17:e0275762. doi: 10.1371/journal.pone.0275762, PMID: 36215219 PMC9551627

[ref6] Al-Talib H. Zuraina N. Kamarudin B. Yean C. Y. (2015). Genotypic variations of virulent genes in Enterococcus faecium and *Enterococcus faecalis* isolated from three hospitals in Malaysia. Adv. Clin. Exp. Med. 24, 121–127. doi: 10.17219/acem/38162, PMID: 25923096

[ref7] Bai J. Qiao X. Ma Y. Han M. Jia S. Huang X.. (2020). Protection efficacy of Oral bait probiotic vaccine constitutively expressing tetravalent toxoids against *Clostridium perfringens* exotoxins in livestock (rabbits). Vaccines (Basel) 8:17. doi: 10.3390/vaccines8010017, PMID: 31936328 PMC7157649

[ref8] Ballesté-Delpierre C. Fernandez-Orth D. Ferrer-Navarro M. Díaz-Peña R. Odena-Caballol A. Oliveira E.. (2017). First insights into the pleiotropic role of vrf (yedF), a newly characterized gene of *Salmonella Typhimurium*. Sci. Rep. 7:15291. doi: 10.1038/s41598-017-15369-7, PMID: 29127378 PMC5681696

[ref9] Benoit S. L. Schmalstig A. A. Glushka J. Maier S. E. Edison A. S. Maier R. J. (2019). Nickel chelation therapy as an approach to combat multi-drug resistant enteric pathogens. Sci. Rep. 9:13851. doi: 10.1038/s41598-019-50027-0, PMID: 31554822 PMC6761267

[ref10] Bin Masalam M. S. Bahieldin A. Alharbi M. G. Al-Masaudi S. Al-Jaouni S. K. Harakeh S. M.. (2018). Isolation, molecular characterization and probiotic potential of lactic acid Bacteria in Saudi raw and fermented Milk. Evid. Based Complement. Alternat. Med. 2018, 7970463–7970412. doi: 10.1155/2018/7970463, PMID: 30147735 PMC6083559

[ref11] Bojanic Rasovic M. Mayrhofer S. Martinovic A. Dürr K. Domig K. J. (2017). Lactococci of local origin as potential starter cultures for traditional Montenegrin cheese production. Food Technol. Biotechnol. 55, 55–66. doi: 10.17113/ftb.55.01.17.4854, PMID: 28559734 PMC5434366

[ref12] Campagne J. Guichard J. F. Moulhade M. C. Kawski H. Maurier F. (2020). Lactobacillus endocarditis: a case report in France and literature review. IDCases 21:e00811. doi: 10.1016/j.idcr.2020.e00811, PMID: 32477869 PMC7248674

[ref13] Cockerill F. (2012). Performance Standards for Antimicrobial Susceptibility Testing: Twenty-Second Informational Supplement [Provides Updated Tables for M02-A11 and M07-A9]. Wayne, PA: Clinical and Laboratory Standards Institute.

[ref14] Colautti A. Arnoldi M. Comi G. Iacumin L. (2022). Antibiotic resistance and virulence factors in lactobacilli: something to carefully consider. Food Microbiol. 103:103934. doi: 10.1016/j.fm.2021.103934, PMID: 35082060

[ref15] Collado M. C. Grześkowiak Ł. Salminen S. (2007). Probiotic strains and their combination inhibit in vitro adhesion of pathogens to pig intestinal mucosa. Curr. Microbiol. 55, 260–265. doi: 10.1007/s00284-007-0144-8, PMID: 17657533

[ref16] Connil N. Le Breton Y. Dousset X. Auffray Y. Rincé A. Prévost H. (2002). Identification of the *Enterococcus faecalis* tyrosine decarboxylase operon involved in tyramine production. Appl. Environ. Microbiol. 68, 3537–3544. doi: 10.1128/aem.68.7.3537-3544.2002, PMID: 12089039 PMC126796

[ref17] Dowarah R. Verma A. K. Agarwal N. Singh P. Singh B. R. (2018). Selection and characterization of probiotic lactic acid bacteria and its impact on growth, nutrient digestibility, health and antioxidant status in weaned piglets. PLoS One 13:e0192978. doi: 10.1371/journal.pone.0192978, PMID: 29518093 PMC5843174

[ref9002] FAO/WHO. (2002). Food and Agriculture Organization - World Health Organization. Report of a Joint FAOWHO Working Group on Drafting Guidelines for the Evaluation of Probiotics in Food. Geneva: WHO.

[ref18] Fernández M. del Río B. Linares D. M. Martín M. C. Alvarez M. A. (2006). Real-time polymerase chain reaction for quantitative detection of histamine-producing bacteria: use in cheese production. J. Dairy Sci. 89, 3763–3769. doi: 10.3168/jds.S0022-0302(06)72417-1, PMID: 16960050

[ref19] Fontana L. Bermudez-Brito M. Plaza-Diaz J. Muñoz-Quezada S. Gil A. (2013). Sources, isolation, characterisation and evaluation of probiotics. Br. J. Nutr. 109, S35–S50. doi: 10.1017/s0007114512004011 23360880

[ref20] Gao J. Cao S. Xiao H. Hu S. Yao K. Huang K.. (2022). *Lactobacillus reuteri* 1 enhances intestinal epithelial barrier function and alleviates the inflammatory response induced by Enterotoxigenic *Escherichia coli* K88 via suppressing the MLCK signaling pathway in IPEC-J2 cells. Front. Immunol. 13:897395. doi: 10.3389/fimmu.2022.897395, PMID: 35911699 PMC9331657

[ref21] Ghattargi V. C. Gaikwad M. A. Meti B. S. Nimonkar Y. S. Dixit K. Prakash O.. (2018). Comparative genome analysis reveals key genetic factors associated with probiotic property in *Enterococcus faecium* strains. BMC Genomics 19:652. doi: 10.1186/s12864-018-5043-9, PMID: 30180794 PMC6122445

[ref22] Gheziel C. Russo P. Arena M. P. Spano G. Ouzari H. I. Kheroua O.. (2019). Evaluating the probiotic potential of *Lactobacillus plantarum* strains from Algerian infant feces: towards the Design of Probiotic Starter Cultures Tailored for developing countries. Probiotics Antimicrob. Proteins 11, 113–123. doi: 10.1007/s12602-018-9396-9, PMID: 29460213

[ref23] Gokhale S. Bhaduri A. (2019). Provitamin D (3) modulation through prebiotics supplementation: simulation based assessment. Sci. Rep. 9:19267. doi: 10.1038/s41598-019-55699-2, PMID: 31848400 PMC6917722

[ref24] Gómez N. C. Ramiro J. M. Quecan B. X. de Melo Franco B. D. (2016). Use of potential probiotic lactic acid Bacteria (LAB) biofilms for the control of *Listeria monocytogenes*, salmonella typhimurium, and *Escherichia coli* O157:H7 biofilms formation. Front. Microbiol. 7:863. doi: 10.3389/fmicb.2016.00863, PMID: 27375584 PMC4901071

[ref25] Guo L. Li T. Tang Y. Yang L. Huo G. (2016). Probiotic properties of Enterococcus strains isolated from traditional naturally fermented cream in China. Microb. Biotechnol. 9, 737–745. doi: 10.1111/1751-7915.12306, PMID: 26200795 PMC5072190

[ref26] Hamon E. Horvatovich P. Izquierdo E. Bringel F. Marchioni E. Aoudé-Werner D.. (2011). Comparative proteomic analysis of *Lactobacillus plantarum* for the identification of key proteins in bile tolerance. BMC Microbiol. 11:63. doi: 10.1186/1471-2180-11-63, PMID: 21447177 PMC3073879

[ref27] He Q. Li J. Ma Y. Chen Q. Chen G. (2021). Probiotic potential and cholesterol-lowering capabilities of bacterial strains isolated from Pericarpium Citri Reticulatae “Chachiensis”. Microorganisms 9:1224. doi: 10.3390/microorganisms9061224, PMID: 34200041 PMC8227569

[ref28] Hirano S. Yokota Y. Eda M. Kuda T. Shikano A. Takahashi H.. (2017). Effect of *Lactobacillus plantarum* Tennozu-SU2 on *Salmonella Typhimurium* infection in human enterocyte-like HT-29-Luc cells and BALB/c mice. Probiotics Antimicrob. Proteins 9, 64–70. doi: 10.1007/s12602-016-9243-9, PMID: 27943051

[ref29] Hsu T. C. Yi P. J. Lee T. Y. Liu J. R. (2018). Probiotic characteristics and zearalenone-removal ability of a *Bacillus licheniformis* strain. PLoS One 13:e0194866. doi: 10.1371/journal.pone.0194866, PMID: 29641608 PMC5895015

[ref30] Iñiguez-Palomares C. Jiménez-Flores R. Vázquez-Moreno L. Ramos-Clamont-Montfort G. Acedo-Félix E. (2011). Protein-carbohydrate interactions between Lactobacillus salivarius and pig mucins. J. Anim. Sci. 89, 3125–3131. doi: 10.2527/jas.2010-2996 21622872

[ref31] Jäger R. Purpura M. Farmer S. Cash H. A. Keller D. (2018). Probiotic *Bacillus coagulans* GBI-30, 6086 improves protein absorption and utilization. Probiotics Antimicrob. Proteins 10, 611–615. doi: 10.1007/s12602-017-9354-y, PMID: 29196920 PMC6208742

[ref32] Jose N. M. Bunt C. R. Hussain M. A. (2015). Comparison of microbiological and probiotic characteristics of lactobacilli isolates from dairy food products and animal rumen contents. Microorganisms 3, 198–212. doi: 10.3390/microorganisms3020198, PMID: 27682086 PMC5023236

[ref33] Kiššová Z. Tkáčiková Ľ. Mudroňová D. Bhide M. R. (2022). Immunomodulatory effect of *Lactobacillus reuteri* (Limosilactobacillus reuteri) and its exopolysaccharides investigated on epithelial cell line IPEC-J2 challenged with *Salmonella Typhimurium*. Life (Basel) 12:1955. doi: 10.3390/life12121955, PMID: 36556320 PMC9788328

[ref34] König E. Sali V. Heponiemi P. Salminen S. Valros A. Junnikkala S.. (2021). Herd-level and individual differences in fecal lactobacilli dynamics of growing pigs. Animals (Basel) 11:113. doi: 10.3390/ani11010113, PMID: 33430499 PMC7827896

[ref35] Kuppusamy P. Kim D. Soundharrajan I. Park H. S. Jung J. S. Yang S. H.. (2020). Low-carbohydrate tolerant LAB strains identified from rumen fluid: investigation of probiotic activity and legume silage fermentation. Microorganisms 8:1044. doi: 10.3390/microorganisms8071044, PMID: 32674395 PMC7409070

[ref36] Landete J. M. (2017). A review of food-grade vectors in lactic acid bacteria: from the laboratory to their application. Crit. Rev. Biotechnol. 37, 296–308. doi: 10.3109/07388551.2016.1144044, PMID: 26918754

[ref37] Li M. Wang Y. Cui H. Li Y. Sun Y. Qiu H. J. (2020). Characterization of lactic acid Bacteria isolated from the gastrointestinal tract of a wild boar as potential probiotics. Front. Vet. Sci. 7:49. doi: 10.3389/fvets.2020.00049 32118070 PMC7026679

[ref38] Ling Z. Cheng Y. Yan X. Shao L. Liu X. Zhou D.. (2020). Alterations of the fecal microbiota in Chinese patients with multiple sclerosis. Front. Immunol. 11:590783. doi: 10.3389/fimmu.2020.590783, PMID: 33391265 PMC7772405

[ref39] Lukic J. Strahinic I. Milenkovic M. Nikolic M. Tolinacki M. Kojic M.. (2014). Aggregation factor as an inhibitor of bacterial binding to gut mucosa. Microb. Ecol. 68, 633–644. doi: 10.1007/s00248-014-0426-1, PMID: 24823989

[ref40] Mao Y. Zhang X. Xu Z. (2020). Identification of antibacterial substances of *Lactobacillus plantarum* DY-6 for bacteriostatic action. Food Sci. Nutr. 8, 2854–2863. doi: 10.1002/fsn3.1585, PMID: 32566203 PMC7300085

[ref41] McBride S. M. Coburn P. S. Baghdayan A. S. Willems R. J. Grande M. J. Shankar N.. (2009). Genetic variation and evolution of the pathogenicity island of *Enterococcus faecalis*. J. Bacteriol. 191, 3392–3402. doi: 10.1128/jb.00031-09, PMID: 19270086 PMC2687173

[ref42] Mehdi Y. Létourneau-Montminy M. P. Gaucher M. L. Chorfi Y. Suresh G. Rouissi T.. (2018). Use of antibiotics in broiler production: global impacts and alternatives. Anim. Nutr. 4, 170–178. doi: 10.1016/j.aninu.2018.03.002 30140756 PMC6103476

[ref43] Miller L. E. Ouwehand A. C. Ibarra A. (2017). Effects of probiotic-containing products on stool frequency and intestinal transit in constipated adults: systematic review and meta-analysis of randomized controlled trials. Ann. Gastroenterol. 30, 629–639. doi: 10.20524/aog.2017.0192, PMID: 29118557 PMC5670282

[ref44] Petrof E. O. (2009). Probiotics and gastrointestinal disease: clinical evidence and basic science. Antiinflamm. Antiallergy Agents Med. Chem. 8, 260–269. doi: 10.2174/187152309789151977, PMID: 20890386 PMC2947383

[ref45] Ran X. He Y. Ai Q. Shi Y. (2021). Effect of antibiotic-induced intestinal dysbacteriosis on bronchopulmonary dysplasia and related mechanisms. J. Transl. Med. 19:155. doi: 10.1186/s12967-021-02794-6, PMID: 33874953 PMC8054697

[ref46] Saboori B. Shahidi F. Hedayati S. Javadmanesh A. (2022). Investigating the probiotic properties and antimicrobial activity of lactic acid Bacteria isolated from an Iranian fermented dairy product, Kashk. Food Secur. 11:3904. doi: 10.3390/foods11233904, PMID: 36496711 PMC9739453

[ref47] Sánchez B. Cobo A. Hidalgo M. Martínez-Rodríguez A. M. Prieto I. Gálvez A.. (2019). Influence of the type of diet on the incidence of pathogenic factors and antibiotic resistance in enterococci isolated from Faeces in mice. Int. J. Mol. Sci. 20:4290. doi: 10.3390/ijms20174290, PMID: 31480694 PMC6747218

[ref48] Sharma K. Attri S. Goel G. (2019). Selection and evaluation of probiotic and functional characteristics of autochthonous lactic acid Bacteria isolated from fermented wheat flour dough Babroo. Probiotics Antimicrob. Proteins 11, 774–784. doi: 10.1007/s12602-018-9466-z, PMID: 30220016

[ref49] Shi Y. Cui X. Gu S. Yan X. Li R. Xia S.. (2019). Antioxidative and probiotic activities of lactic acid Bacteria isolated from traditional artisanal Milk cheese from Northeast China. Probiotics Antimicrob. Proteins 11, 1086–1099. doi: 10.1007/s12602-018-9452-5, PMID: 30056601

[ref50] Talib N. Mohamad N. E. Yeap S. K. Hussin Y. Aziz M. N. M. Masarudin M. J.. (2019). Isolation and characterization of Lactobacillus spp. from kefir samples in Malaysia. Molecules 24:2606. doi: 10.3390/molecules24142606, PMID: 31319614 PMC6680525

[ref51] Valeriano V. D. Parungao-Balolong M. M. Kang D. K. (2014). In vitro evaluation of the mucin-adhesion ability and probiotic potential of *Lactobacillus mucosae* LM1. J. Appl. Microbiol. 117, 485–497. doi: 10.1111/jam.12539, PMID: 24807045

[ref9001] Vahedi Shahandashti R. Kasra Kermanshahi R. Ghadam P. (2016). The inhibitory effect of bacteriocin produced by Lactobacillus acidophilusATCC 4356 and Lactobacillus plantarum ATCC 8014 on planktonic cells and biofilms of Serratia marcescens. Turk J Med Sci. 46, 1188–1196. doi: 10.3906/sag-1505-51 27513424

[ref52] Vankerckhoven V. Van Autgaerden T. Vael C. Lammens C. Chapelle S. Rossi R.. (2004). Development of a multiplex PCR for the detection of asa1, gelE, cylA, esp, and hyl genes in enterococci and survey for virulence determinants among European hospital isolates of *Enterococcus faecium*. J. Clin. Microbiol. 42, 4473–4479. doi: 10.1128/jcm.42.10.4473-4479.2004, PMID: 15472296 PMC522368

[ref53] Vinderola G. Matar C. Perdigón G. (2007). Milk fermented by *Lactobacillus helveticus* R389 and its non-bacterial fraction confer enhanced protection against *Salmonella enteritidis* serovar typhimurium infection in mice. Immunobiology 212, 107–118. doi: 10.1016/j.imbio.2006.09.003, PMID: 17336831

[ref54] Vogado C. O. Leandro E. D. S. Zandonadi R. P. de Alencar E. R. Ginani V. C. Nakano E. Y.. (2018). Enrichment of probiotic fermented Milk with green Banana pulp: characterization microbiological, physicochemical and sensory. Nutrients 10:427. doi: 10.3390/nu10040427, PMID: 29596319 PMC5946212

[ref55] Wang W. Ma H. Yu H. Qin G. Tan Z. Wang Y.. (2020). Screening of *Lactobacillus plantarum* Subsp. plantarum with potential probiotic activities for inhibiting ETEC K88 in weaned piglets. Molecules 25:4481. doi: 10.3390/molecules25194481, PMID: 33003556 PMC7582832

[ref56] Wang M. Wu H. Lu L. Jiang L. Yu Q. (2020). *Lactobacillus reuteri* promotes intestinal development and regulates mucosal immune function in newborn piglets. Front. Vet. Sci. 7:42. doi: 10.3389/fvets.2020.00042, PMID: 32118065 PMC7018766

[ref57] Wang G. Yu Y. Garcia-Gutierrez E. Jin X. He Y. Wang L.. (2019). *Lactobacillus acidophilus* JCM 1132 strain and its mutant with different Bacteriocin-producing behaviour have various in situ effects on the gut microbiota of healthy mice. Microorganisms 8:49. doi: 10.3390/microorganisms8010049, PMID: 31881756 PMC7022661

[ref58] Wu Y. Zhu C. Chen Z. Chen Z. Zhang W. Ma X.. (2016). Protective effects of *Lactobacillus plantarum* on epithelial barrier disruption caused by enterotoxigenic *Escherichia coli* in intestinal porcine epithelial cells. Vet. Immunol. Immunopathol. 172, 55–63. doi: 10.1016/j.vetimm.2016.03.005, PMID: 27032504

[ref59] Yang H. Huang X. Fang S. He M. Zhao Y. Wu Z.. (2017). Unraveling the fecal microbiota and metagenomic functional capacity associated with feed efficiency in pigs. Front. Microbiol. 8:1555. doi: 10.3389/fmicb.2017.01555, PMID: 28861066 PMC5559535

[ref60] Yu Z. Zhang X. Li S. Li C. Li D. Yang Z. (2013). Evaluation of probiotic properties of *Lactobacillus plantarum* strains isolated from Chinese sauerkraut. World J. Microbiol. Biotechnol. 29, 489–498. doi: 10.1007/s11274-012-1202-3, PMID: 23117677

[ref61] Zhang X. Hu Y. Ansari A. R. Akhtar M. Chen Y. Cheng R.. (2022). Caecal microbiota could effectively increase chicken growth performance by regulating fat metabolism. Microb. Biotechnol. 15, 844–861. doi: 10.1111/1751-7915.13841, PMID: 34264533 PMC8913871

[ref62] Zhang W. Liu M. Dai X. (2013a). Biological characteristics and probiotic effect of *Leuconostoc lactis* strain isolated from the intestine of black porgy fish. Braz. J. Microbiol. 44, 685–691. doi: 10.1590/s1517-83822013005000053, PMID: 24516418 PMC3910175

[ref63] Zhang H. Ma W. Sun Z. Zhu C. Werid G. M. Ibrahim Y. M.. (2021). Abundance of Lactobacillus in porcine gut microbiota is closely related to immune response following PRRSV immunization. Vet. Microbiol. 259:109134. doi: 10.1016/j.vetmic.2021.109134, PMID: 34087673

[ref64] Zhang M. Shi M. Fan M. Xu S. Li Y. Zhang T.. (2018). Comparative analysis of gut microbiota changes in Père David's deer populations in Beijing Milu Park and Shishou, Hubei Province in China. Front. Microbiol. 9:1258. doi: 10.3389/fmicb.2018.01258, PMID: 29946310 PMC6005820

[ref65] Zhang W. Wang H. Liu J. Zhao Y. Gao K. Zhang J. (2013b). Adhesive ability means inhibition activities for lactobacillus against pathogens and S-layer protein plays an important role in adhesion. Anaerobe 22, 97–103. doi: 10.1016/j.anaerobe.2013.06.005, PMID: 23792230

[ref66] Zhong Y. Fu D. Deng Z. Tang W. Mao J. Zhu T.. (2022). Lactic acid Bacteria mixture isolated from wild pig alleviated the gut inflammation of mice challenged by *Escherichia coli*. Front. Immunol. 13:822754. doi: 10.3389/fimmu.2022.822754, PMID: 35154141 PMC8825813

[ref67] Zommiti M. Connil N. Hamida J. B. Ferchichi M. (2017). Probiotic characteristics of *Lactobacillus curvatus* DN317, a strain isolated from chicken ceca. Probiotics Antimicrob. Proteins 9, 415–424. doi: 10.1007/s12602-017-9301-y, PMID: 28741151

