# Supplementary figures and images for: Molecular identification and probiotic potential characterization of lactic acid bacteria isolated from the pigs with superior immune responses
Source: Front Microbiol. 2024 Mar 21;15:1361860. doi: 10.3389/fmicb.2024.1361860 (PMC10995931; doi:10.3389/fmicb.2024.1361860)

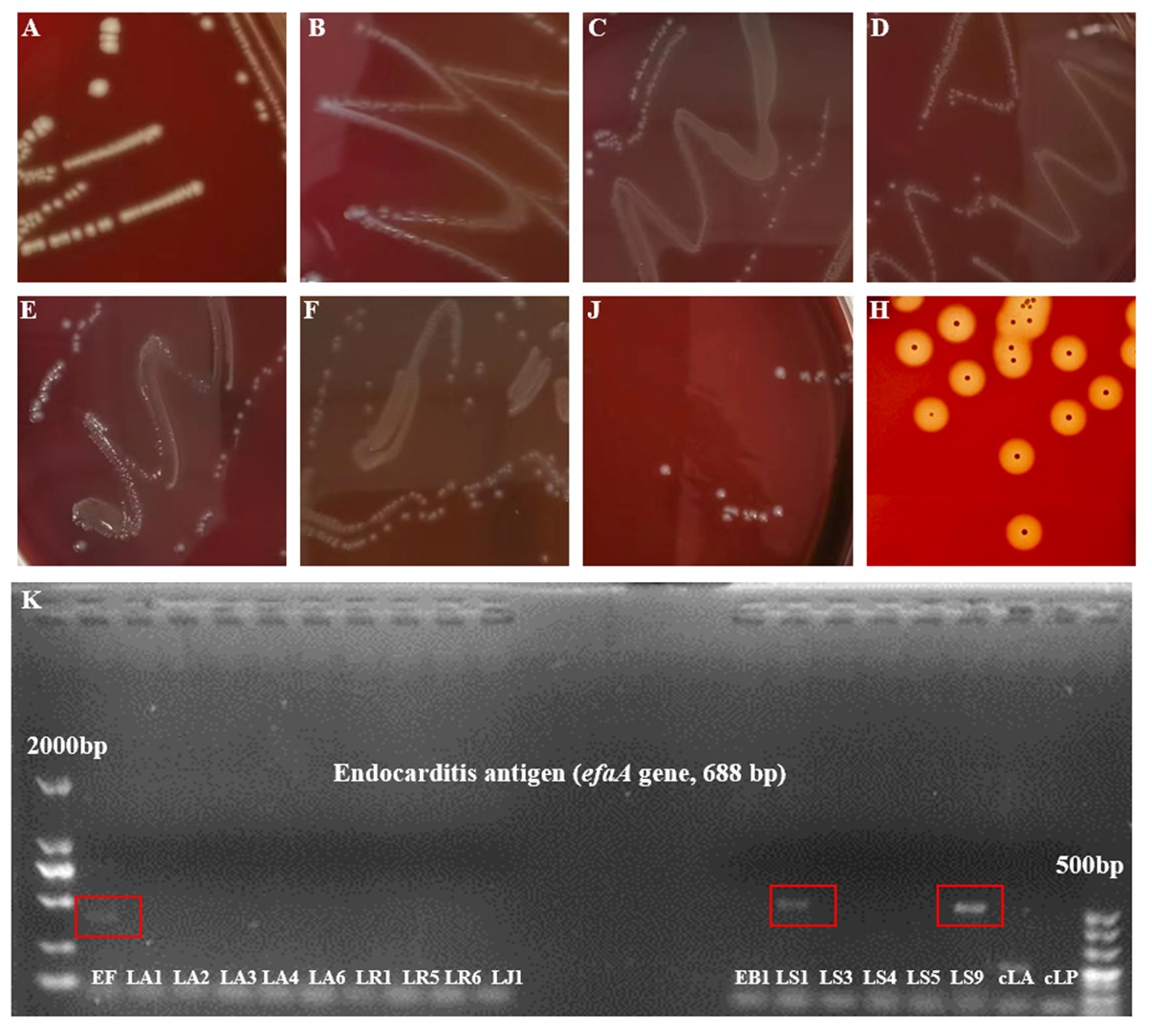

Supplement: Supplementary file 1 [file Image_1.JPEG]
